# HCC-derived SNU cell lines as model systems to study HBV life cycle

**DOI:** 10.1128/jvi.01144-25

**Published:** 2025-09-25

**Authors:** Igor Zaiets, Oleksandra Chazova, Sumedha Gunewardena, Severin O. Gudima

**Affiliations:** 1 Department of Microbiology, Molecular Genetics and Immunology, University of Kansas Medical Center 21638, Kansas City, Kansas, USA; 2 Department of Molecular and Integrative Physiology, University of Kansas Medical Center, Kansas City, Kansas, USA; Wake Forest University School of Medicine, Winston-Salem, North Carolina, USA

**Keywords:** chronic HBV infection, HBV-associated HCC, markers of HBV genome replication, HBV RNA types, integrant-transcribed HBV RNAs, replication-derived HBV RNAs

## Abstract

Human SNU cell lines, derived from hepatocellular carcinomas associated with chronic hepatitis B virus (HBV) infection, were examined. The analysis of intracellular RNA and DNA markers of HBV replication and examination of HBV RNA readss coverage of selected regions on HBV-related RNAs and polyadenylation positions within HBV sequence using RNA-sequencing suggested the absence of HBV replication in SNU-423, SNU-368, SNU-398, SNU-182, SNU-449, SNU-475, SNU-354, SNU-739, and SNU-387 cells, while SNU-761 and SNU-886 still could maintain residual HBV replication. The undetectable intracellular HBV core antigen (HBcAg) and absence of significant levels of secreted core-associated and virion-associated HBV DNA confirmed the absence or profound suppression of HBV replication in parental SNU cell lines. Various 5′-human-HBV-3′ and 5′-HBV-human-3′ RNAs transcribed from integrated HBV DNA were found in most of the SNU cell lines. The 5′-HBV-human-3′ junctions suggested that several SNU cell lines could generate 5′-HBV-human-3′ RNAs encoding HBV envelope proteins. The known and novel spliced HBV RNAs were detected in SNU-886, SNU-739, SNU-387, SNU-761, and SNU-354 cells. At least some of them were generated independently of HBV replication. None of the SNU cell lines supported efficient HBV replication after transfection with the vector that initiates efficient HBV replication in Huh7 cells. This was reflected by three distinct accumulation patterns of HBV replication markers, undetectable intracellular HBcAg, and by the lack of considerable levels of secreted core-bound and virion-associated HBV DNA. Overall, SNU cell lines represent valuable model systems for detailed analysis of integrant-transcribed HBV RNAs, spliced HBV RNAs, and mechanisms of suppression of HBV genome replication.

**IMPORTANCE:**

SNU cell lines without ongoing hepatitis B virus (HBV) genome replication are invaluable experimental systems that allow detailed study of the biogenesis and properties of integrant-transcribed 5′-human-HBV-3′ and 5′-HBV-human-3′ RNAs and mechanisms generating spliced HBV-related RNA species independently of concomitant viral replication. Three unique patterns of intracellular accumulation of HBV replication markers were observed in SNU cell lines transfected with the vector that initiates efficient HBV genome replication in Huh7 cells: (i) very low levels of pre-genomic RNA (pgRNA), total HBV RNA, replication-derived RNAs (rd-RNAs), covalently closed circular DNA (cccDNA), and core-associated HBV DNA; (ii) moderate pgRNA, high total HBV RNA, rd-RNAs, and cccDNA, but very low core-associated HBV DNA; and (iii) very low pgRNA, total HBV RNA, rd-RNAs, and core-associated HBV DNA, but moderate/high cccDNA likely reflect three natural host-mediated mechanisms suppressing HBV replication, the analysis of which should advance our understanding of HBV-host interactions and could be informative for the search for novel anti-HBV interventions.

## INTRODUCTION

Hepatitis B virus (HBV) remains a significant human pathogen with approximately 300 million individuals chronically infected with HBV worldwide (1–5). HBV is a small DNA virus. The viral genome is a partially double-stranded relaxed circular DNA (rcDNA), which is encapsidated inside the nucleocapsid that is coated with the viral envelope proteins or surface antigen (HBsAg). The minus strand of rcDNA is complete, while the plus strand is incomplete, and none of the strands are chemically ligated.

Using receptor-mediated endocytosis, HBV enters the susceptible cells. After the intracellular uncoating, when the virus loses its envelope that mostly consists of the viral envelope proteins, the DNA synthesis inside the nucleocapsid (or core) resumes, and the plus strand gets completed. After the capsid entry into the nucleus and its disassembly, the viral DNA genome gets converted into the covalently closed circular DNA (cccDNA), which is the template for the transcription of all viral RNA species produced by HBV replication. The pre-genomic RNA (pgRNA) is the mRNA for the production of the viral core protein and polymerase. HBV replicates through reverse transcription that starts when the complex of the polymerase and pgRNA is assembled into the capsid. The pgRNA is the template for reverse transcription. The major end product of the reverse transcription is rcDNA. The capsid bearing rcDNA can either be enveloped and secreted as a mature HBV virion or it can be transported back to the nucleus to replenish the cccDNA pool.

An alternative product of the reverse transcription is the double-stranded linear DNA genome or DSL DNA of HBV. The DSL is made when the RNA primer is not translocated during the initiation of plus-strand DNA synthesis, which leads to the production of a viral linear DNA intermediate that differs from rcDNA. The DSL is the main substrate of HBV DNA integration, which is random and is facilitated by the host cellular DNA repair enzymes (1, 2, 6–8). The progeny virus cannot be produced from the integrated HBV DNA, but a variety of hybrid 5′-human-HBV-3′ RNAs and 5′-HBV-human-3′ RNA species are transcribed from the integrants. Some of the 5′-HBV-human-3' RNAs are capable of producing HBV envelope proteins independently of HBV genome replication. The above-mentioned integrant-transcribed HBV-related RNA species (5′-human-HBV-3′ RNAs and 5′-HBV-human-3′ RNAs) remain understudied (9–14).

HBV infects mature differentiated liver hepatocytes and is able to induce transient and chronic HBV infection. A considerable number of individuals who clinically resolved HBV infection still maintain a residual HBV reservoir in their livers that is controlled by the immune system. Chronic HBV infection is still the main risk factor for developing hepatocellular carcinoma (HCC). More than 50% of all known HCC cases are associated with chronic HBV infection. Currently, there are no curative drugs for HBV. The anti-HBV treatment using nucleos(t)ide analogs is a lifelong therapy. The therapy stoppage usually results in the HBV rebound to pre-treatment levels (1–4, 15–21). The mechanisms of the replication and pathogenesis of HBV are far from being fully understood.

One important category of the experimental systems used in the HBV research field is represented by human cell lines derived from HBV-related HCCs. A number of such cell lines were developed by Seoul National University (SNU), and they are called SNU cell lines. These SNU cell lines were originated from primary HCCs harvested from Korean patients chronically infected with HBV. They all were adherent cell lines, and most of the cells in culture appeared to maintain many of the morphological features of the HCCs, from which the SNU cell lines were developed. It was reported that the doubling time for SNU cell lines was in the range of 34–72 h. It was also noted that SNU cell lines contained integrated HBV DNA, and for some of the SNU cell lines, the presence of RNAs derived from integrated HBV DNA was confirmed via RT-PCR assays. It was found that the cell lines SNU-368, SNU-449, SNU-398, SNU-182, and SNU-475 each had unique modal karyotypic features and variable structural and numerical clonal cytogenetic aberrations. While SNU cell lines, originated from HCCs collected from Korean individuals chronically infected with HBV, have been available for researchers for more than two decades, they have not been extensively characterized for the presence of HBV markers and for the status of HBV genome replication in them (22–26).

This study examined in detail 11 different SNU cell lines produced from HBV-related HCCs. By analyzing intracellular HBV replication markers, pgRNA, replication-derived RNAs transcribed from cccDNA and polyadenylated using the HBV conventional polyadenylation signal (rd-RNAs), total HBV RNA, core-associated HBV DNA, and cccDNA, as well as investigating the intracellular expression of HBV core antigen (HBcAg) and analyzing secreted HBV DNA associated with either unenveloped nucleocapsids or the virions coated with HBV envelope proteins, and also by examining HBV-related RNA species accumulated in the SNU cell lines, we determined that at least 9 of 11 parental SNU cell lines (SNU-423, SNU-368, SNU-398, SNU-182, SNU-449, SNU-475, SNU-354, SNU-739, and SNU-387) did not have an ongoing HBV genome replication in them. However, SNU-761 and SNU-886 could still have some residual levels of HBV genome replication. At the same time, many of the SNU cell lines accumulated both types of the integrant-transcribed HBV RNAs, 5′-human-HBV-3′ RNAs and 5′-HBV-human-3′ RNAs. The analysis of the viral-host junction sites suggested that a portion of 5′-HBV-human-3′ RNAs could likely serve as mRNAs for the HBV envelope proteins (or surface antigen, HBsAg). We also found known and novel variants of spliced HBV-related RNAs, of which at least some represent spliced integrant-transcribed RNAs generated in the absence of HBV genome replication.

Furthermore, we observed that after transfection with the plasmid pT-HBV1.3 (27), which initiates efficient HBV replication in Huh7 cells and HepG2 cells, the transfected SNU cell lines were unable to support efficient HBV genome replication as compared to Huh7 cells and HepG2 cells. This observation is consistent with the hypothesis that during chronic HBV infection, livers get repopulated with hepatocytes that poorly support HBV replication, and that some of these hepatocytes can acquire cancer-related mutation(s) and give rise to clonal HCCs. The available data suggest that HBV-related HCCs often originated from hepatocytes poorly replicating HBV (6–8, 28–32). Interestingly, three distinct patterns of accumulation of HBV replication markers reflecting the deficiency of SNU cell lines in terms of the support of HBV replication were observed in transfected SNU cell lines: (i) very low levels of pgRNA, total HBV RNA, rd-RNAs, cccDNA, and core-associated DNA; (ii) moderate pgRNA, high total HBV RNA, rd-RNAs, and cccDNA, but very low core-associated DNA; and (iii) very low pgRNA, total HBV RNA, rd-RNAs, and core-associated DNA, but moderate/high cccDNA. These results suggest that the host could likely efficiently suppress HBV replication *in vivo* during natural chronic HBV infection by at least three different mechanisms, which affect the accumulation of the above-mentioned HBV replication markers in different and specific ways.

In summary, the analyzed SNU cell lines therefore represent important experimental systems that can be used for detailed analysis of the (i) origination and properties of integrant-transcribed HBV RNAs, (ii) mechanisms generating various spliced HBV-related RNAs in the absence of the HBV genome replication, and (iii) mechanisms of efficient natural suppression of HBV genome replication. The latter should considerably advance our understanding of critical HBV-host interactions regulating the HBV life cycle *in vivo*.

## RESULTS

### Replication markers of HBV in parental SNU cell lines

**Fig 1:**
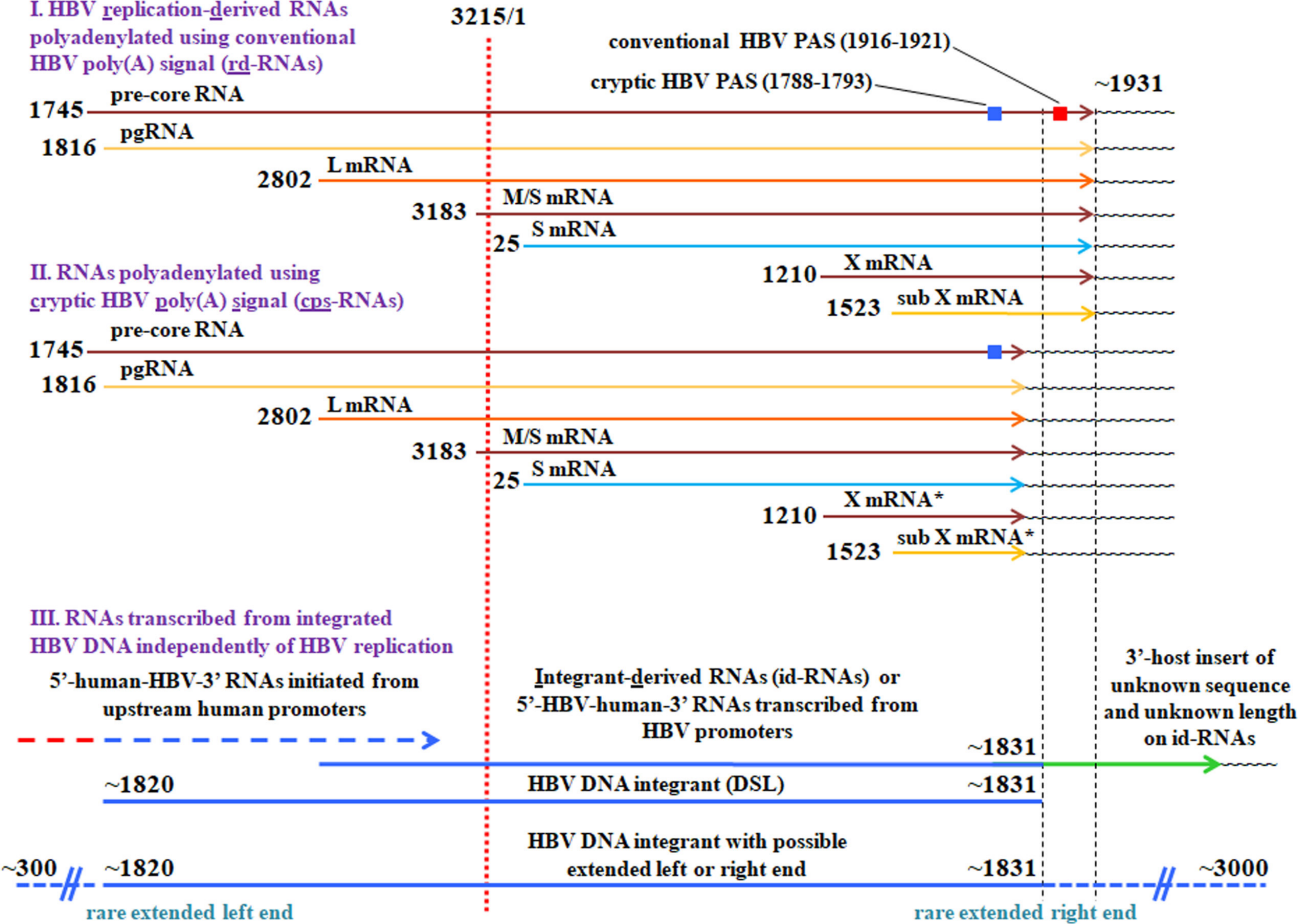
HBV RNAs accumulated during the HBV life cycle. The figure is based on our previously published work containing the representation of diverse HBV RNA species produced during chronic HBV infection, which was summarized in Fig. 1 of reference 12. There are two sources of HBV RNA transcripts during natural HBV infection—HBV genome replication and transcription from integrated HBV DNA that occurs independently of HBV genome replication. The first group of HBV RNAs consists of cccDNA-transcribed RNA species that are generated during the replication of the viral genome and are polyadenylated by employing HBV conventional polyadenylation signal. These RNAs are also known as the replication-derived HBV RNAs or rd-RNAs. This group consists of the pre-core RNA, pre-genomic RNA, three RNAs encoding HBV envelope proteins, and also X mRNA and SubX mRNA (1, 12, 33). The pre-core and pgRNA are longer than the HBV genomic length, and therefore, they contain redundant sequences at their 5′-end and 3′-end. Other rd-RNAs are shorter than the HBV genomic length. The L mRNA encodes Large (L) envelope protein. The M/S mRNA encodes Middle (M) envelope protein. When the first AUG codon (for the M envelope protein) is not in use, M/S mRNA can generate Small (S) envelope protein. The S mRNA encodes the S envelope protein. The X mRNA encodes viral X protein. The SubX mRNA is initiated within the X open reading frame (ORF) and has a shorter 5′-end than the X mRNA. The production of the potential X-related protein that is missing some of the X N-terminal sequences and encoded by the SubX mRNA has not been shown yet. All rd-RNAs share the same 3′-end sequences and usually have their polyadenylation sites within the area of the HBV positions from 1,929 to 1,953 downstream of the conventional HBV PAS (12). The positions of the HBV genome end and start (3,215/1) are indicated as the reference using the coordinates of the HBV reference sequence used, full-length HBV genome GQ358158. The RNAs of the first group are originated only from HBV genome replication and are not transcribed from integrated HBV DNA. The second group consists of HBV RNAs polyadenylated using the viral cryptic PAS also known as cps-RNAs. Since cryptic PAS can remain intact and in the correct position within the integrated HBV DNA, cps-RNAs can be generated by transcription from cccDNA during HBV genome replication and also can be transcribed from HBV DNA integrants independently of the viral replication. These RNAs also share their 3′-ends. However, their 3′-ends are shorter than the 3′-ends of their rd-RNA counterparts. The cps-RNAs are missing a bit more than 100 nucleotides from their 3′-end region compared to rd-RNAs. Because of this, only pre-core RNA, pgRNA, and three HBsAg mRNAs in the group of cps-RNAs still bear their intact ORFs just as the corresponding rd-RNAs. The X and SubX mRNAs in the cps-RNAs group, however, lost the 3′-regions from their ORFs and cannot produce full-length X or putative SubX protein (12, 34). The anticipated 5′-ends (transcription start sites) for rd-RNAs and cps-RNAs are shown at the left-hand side. The third group is represented by two types of integrant-transcribed HBV RNAs that are generated from integrated HBV DNA independently of HBV genome replication. These RNAs are 5′-human-HBV-3′ RNAs, which were initiated from human promoters upstream of integrated HBV DNA, then crossed into HBV sequence and got polyadenylated within the integrated HBV DNA boundaries. There are also 5′-HBV-human-3′ RNAs that were transcribed from HBV promoters in the integrant, copied some HBV sequences, crossed through the HBV/host junction, and got polyadenylated in the downstream human sequence using human PAS. These RNAs are also known as integrant-derived RNAs or id-RNAs. They carry the 3′-end insert of the human sequence of unknown nucleotide sequence and unknown length, reflecting the random nature of HBV DNA integration. The traditional double-stranded linear DNA genome of HBV (DSL HBV DNA), which is the main substrate of the HBV integration, is shown as the reference, and it displays the anticipated left end at the HBV position ~1,820 and the right end at position ~1,831. Therefore, the area between positions 1,831 and 1,931 is not expected to be present on 3′-ends of id-RNAs and is expected to be almost exclusively present on 3′-ends of rd-RNAs. However, on infrequent occasions, there were reported integrants that had their left end protruded much upstream of the position 1,820, and also integrants with their right end extended much further beyond the position 1,831. We had such encounters as well during the analysis of integrant-transcribed HBV RNAs (see Table 3). At the bottom, therefore, is shown the schematic representation of HBV DNA integrants with the occasional (rare) extended left or right end, which indicates the left end of integrated DNA extended upstream up to HBV position ~300 and also possible extended right end downstream to the HBV position ~3,000 (7, 8).

Five different markers of intracellular HBV genome replication have been analyzed using RT-qPCR assays for RNA markers and qPCR assays for DNA markers: (i) pre-core/pgRNA, (ii) rd-RNAs, (iii) total HBV RNA (this PCR spans the amplicon of the HBV positions 1,662 to 1,743 and will amplify the sequences of pre-core RNA, pgRNA, L mRNA, M/S mRNA, S mRNA, X mRNA, and SubX mRNA that were polyadenylated via conventional or cryptic polyadenylation signal [PAS]), and thus it is supposed to detect rd-RNAs and cps-RNAs (HBV RNAs polyadenylated via HBV cryptic PAS [9, 12]) and also various RNA species transcribed from integrated HBV DNA depending on the HBV area that they will bear (Fig. 1), (iv) core-associated HBV DNA, and (v) cccDNA. The results of the measurements are shown in Table 1. The 11 SNU cell lines examined in this study were not previously extensively evaluated in terms of the status of HBV genome replication (22–26). It remains unclear which of these cell lines are free of replicating HBV genomes and which ones may still have some levels of HBV genome replication.

**TABLE 1:** Intracellular HBV replication markers in parental SNU cell lines^*c*^

HBV replication markers^*a*^										
SNU cell line	pgRNA		Total HBV RNA		rd-RNAs		Core-associated HBV DNA		cccDNA	
	Copy numbers/ average cell	SD	Copy numbers/ average cell	SD	Copy numbers/ average cell	SD	Copy numbers/ average cell	SD	Copy numbers/ average cell	SD
SNU-886	9.26	1.31	116.47	13.51	12.16	0.71	4.50 × 10^−6^	6.36 × 10^−6^	0.26	5.99 × 10^−2^
SNU-739	0.32	3.62 × 10^−2^	78.26	19.49	0.99	8.01 × 10^−2^	1.20 × 10^−6^	8.40 × 10^−7^	2.18 × 10^−3^	1.71 × 10^−3^
SNU-387	1.76	0.17	4.74	0.69	1.22 × 10^−2^	5.64 × 10^−4^	Und^*b*^	Und	5.06 × 10^−5^	3.71 × 10^−5^
SNU-423	1.67 × 10^−3^	2.18 × 10^−4^	5.29 × 10^−3^	7.49 × 10^−3^	1.69 × 10^−3^	6.17 × 10^−4^	Und	Und	Und	Und
SNU-761	4.23	0.72	389.70	69.41	20.62	3.24	Und	Und	1.63 × 10^−4^	1.21 × 10^−4^
SNU-475	3.75 × 10^−2^	1.63 × 10^−2^	0.89	0.32	5.63 × 10^−3^	1.20 × 10^−3^	Und	Und	3.69 × 10^−3^	2.43 × 10^−3^
SNU-368	9.29 × 10^−2^	3.14 × 10^−2^	38.62	8.82	2.42 × 10^−3^	1.89 × 10^−3^	Und	Und	Und	Und
SNU-354	0.42	0.11	88.47	36.2	3.82 × 10^−3^	3.09 × 10^−3^	2.40 × 10^−7^	3.60 × 10^−7^	3.43 × 10^−4^	9.22 × 10^−5^
SNU-182	2.03 × 10^−2^	2.62 × 10^−3^	28.04	5.16	0.34	5.13 × 10^−2^	1.38 × 10^−6^	1.98 × 10^−6^	4.08 × 10^−5^	2.12 × 10^−5^
SNU-449	6.67 × 10^−3^	3.92 × 10^−4^	44.82	12.40	1.43 × 10^−3^	4.67 × 10^−4^	4.92 × 10^−6^	2.40 × 10^−6^	4.20 × 10^−6^	1.68 × 10^−6^
SNU-398	8.10 × 10^−4^	9.63 × 10^−5^	8.71 × 10^−3^	4.57 × 10^−3^	Und	Und	8.40 × 10^−7^	4.80 × 10^−7^	1.50 × 10^−3^	5.03 × 10^−5^
Hep3B	Und	Und	12.77	0.71	0.13	4.83 × 10^−2^	Und	Und	Und	Und
PLC/PRF/5	3.50	0.45	0.84	9.58 × 10^−2^	0.86	9.63 × 10^−2^	Und	Und	3.99 × 10^−2^	9.33 × 10^−3^

^*a*^ RNA markers are expressed as copy numbers per average cell (25 pg of total RNA equals approximately one cell [35]). DNA markers are expressed as copy numbers per average cell (6 pg of total DNA equals approximately one cell [36]). Our RT-qPCR used to determine pgRNA levels does not discriminate between pgRNA and pre-core RNA. This, however, is a common approach to measure pgRNA, because during HBV genome replication, pgRNA is a major transcript, while pre-core RNA is a minor transcript (9, 12, 33).

^*b*^ Und, undetermined.

^*c*^ The Hep3B and PLC/PRF/5 cell lines derived from human HBV-related HCCs are included as additional control cell lines for comparison. They both are known to bear integrated HBV DNA, and they do not have ongoing HBV replication in them (37–41).

Different SNU cell lines displayed various levels of the measured RNA and DNA markers of HBV replication. It needs to be emphasized that measured signals for the above-mentioned three RNA markers could be originated from HBV genome replication or reflect the levels of HBV-related RNA species transcribed from integrated HBV DNA independently of HBV replication. The amplified signal for the two above-mentioned DNA markers of HBV replication can also arise from ongoing HBV genome replication or, independently of HBV replication, it can reflect the sequences of chromosomal fragments that bear integrated HBV DNA and were present in the final DNA preparations that were analyzed. It is known that DNA isolation procedures that are used for the analysis of core-associated HBV DNA and cccDNA do not guarantee the absence of fragmented chromosomal DNA in the final DNA preparations (42–45). Therefore, the measured levels of HBV replication markers need to be evaluated with caution.

The measured pre-core/pgRNA numbers were low in SNU-423, SNU-398, SNU-449, SNU-182, and SNU-475. Other cell lines, SNU-368, SNU-354, SNU-387, SNU-739, and SNU-761, had considerably higher pre-core/pgRNA numbers, and the SNU-886 cell line displayed the highest levels of pre-core/pgRNA (Table 1). The highest levels of total HBV RNA were found for SNU-761, SNU-886, SNU-739, and SNU-354 cell lines. Somewhat lower numbers were recorded for SNU-368, SNU-182, and SNU-449 cell lines. Considerably lower numbers were observed for SNU-387 and SNU-475. Finally, SNU-423 and SNU-398 had very low total HBV RNA numbers, which could be consistent with the absence of HBV replication in these cell lines. Low levels of rd-RNAs were found in SNU-423, SNU-475, SNU-387, SNU-368, SNU-354, and SNU-449. In the SNU-398 cell line, rd-RNAs were not detected, which was a strong indication of the absence of HBV genome replication. In this cell line, the signals of pre-core/pgRNA, total HBV RNA, and core-associated DNA were low as well. Considerably higher rd-RNAs numbers were observed for SNU-182, SNU-739, SNU-761, and SNU-886. The latter two cell lines had the highest measured levels of rd-RNAs (Table 1). Interestingly, two cell lines, SNU-423 and SNU-368, displayed undetectable core-associated DNA and cccDNA, which strongly suggests that (i) HBV genome replication is absent from these cell lines, and (ii) the measured levels of three HBV RNA markers (pre-core/pgRNA, rd-RNAs, and total HBV RNA) most likely reflect the integrant-transcribed RNAs (Fig. 1) generated independently of HBV replication. Furthermore, the core-associated DNA was also not detected in SNU-387, SNU-761, and SNU-475 cell lines, and it was at very low levels in the remaining cell lines, SNU-886, SNU-739, SNU-354, SNU-182, SNU-449, and SNU-398, which could be consistent with possibly absent or considerably suppressed HBV replication. The measured cccDNA levels were very low in SNU-387, SNU-761, SNU-182, and SNU-449. Somewhat higher cccDNA numbers were found in SNU-739, SNU-475, SNU-398, and SNU-354 cell lines. The highest levels of cccDNA were measured for the SNU-886 cell line (Table 1).

In addition, we also used two human cell lines as references: Hep3B cells and PLC/PRF/5 cells (or Alexander cells), which were derived from HBV-related HCCs and did not show any signs of HBV replication in them (37–41). The levels of HBV replication markers measured in them were well within the range of the levels measured for the SNU cell lines. Interestingly, no pgRNA and cccDNA were found in Hep3B cells, and both Hep3B and Alexander cells had undetectable intracellular core-associated HBV DNA (Table 1).

**Fig 2:**
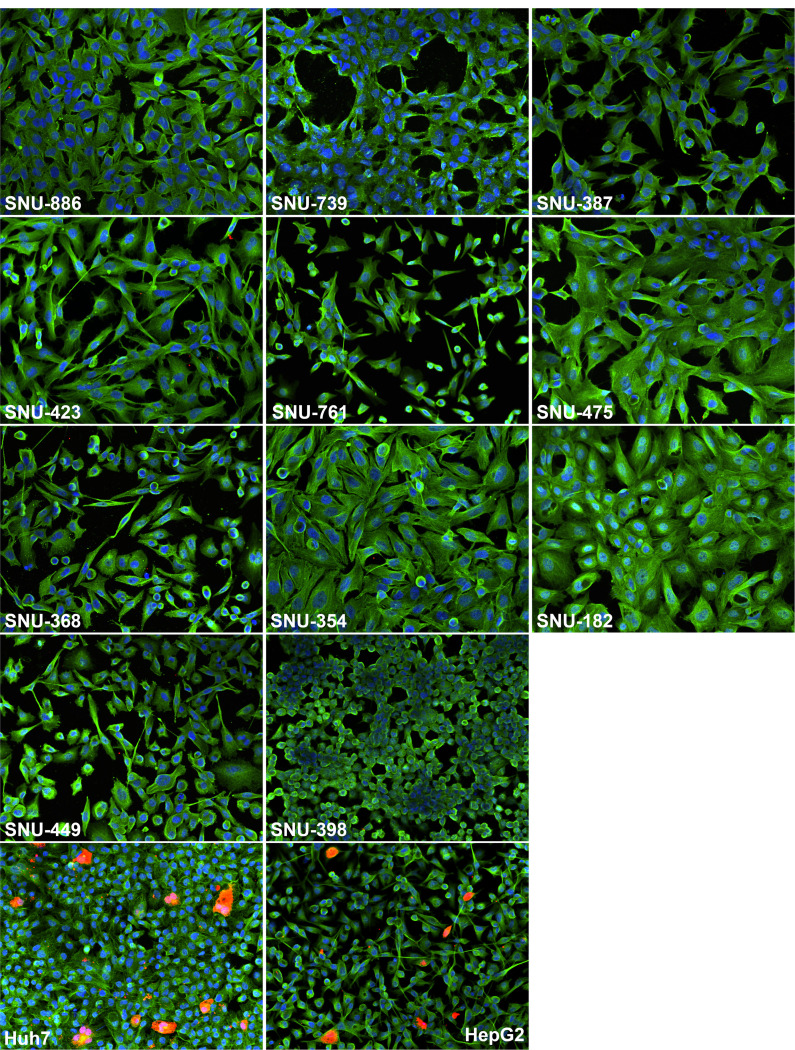
Analysis of intracellular HBcAg expression in parental SNU cell lines using immunofluorescence. Parental SNU cell lines were washed, fixed, permeabilized, and stained for HBcAg (red), beta-tubulin (green), and nuclear DNA (blue) as detailed in Materials and Methods. The control Huh7 cells and HepG2 cells were transfected with pT-HBV1.3, and then on day 8 post-transfection, they were washed, fixed, and stained as it was done for parental SNU cell lines. The names of the cell lines analyzed are indicated on the images.

We next looked for the expression of HBV core antigen (HBcAg) in parental SNU cell lines using immunofluorescence and staining for intracellular HBcAg. As controls, we used Huh7 and HepG2 cells that were replicating HBV after transfection with the plasmid pT-HBV1.3 (Fig. 2). Huh7 and HepG2 cells were derived from human HCCs not related to HBV infection and are known to support efficient HBV genome replication after transfection with a vector expressing pgRNA of HBV (41, 46). As seen from Fig. 2, the expression of HBcAg was only detected in transfected Huh7 and HepG2 cell lines, while no HBcAg-positive cells were found in any of the analyzed 11 parental SNU cell lines. This observation is consistent with the absence or profound suppression of HBV genome replication in the tested parental SNU cell lines.

**Fig 3:**
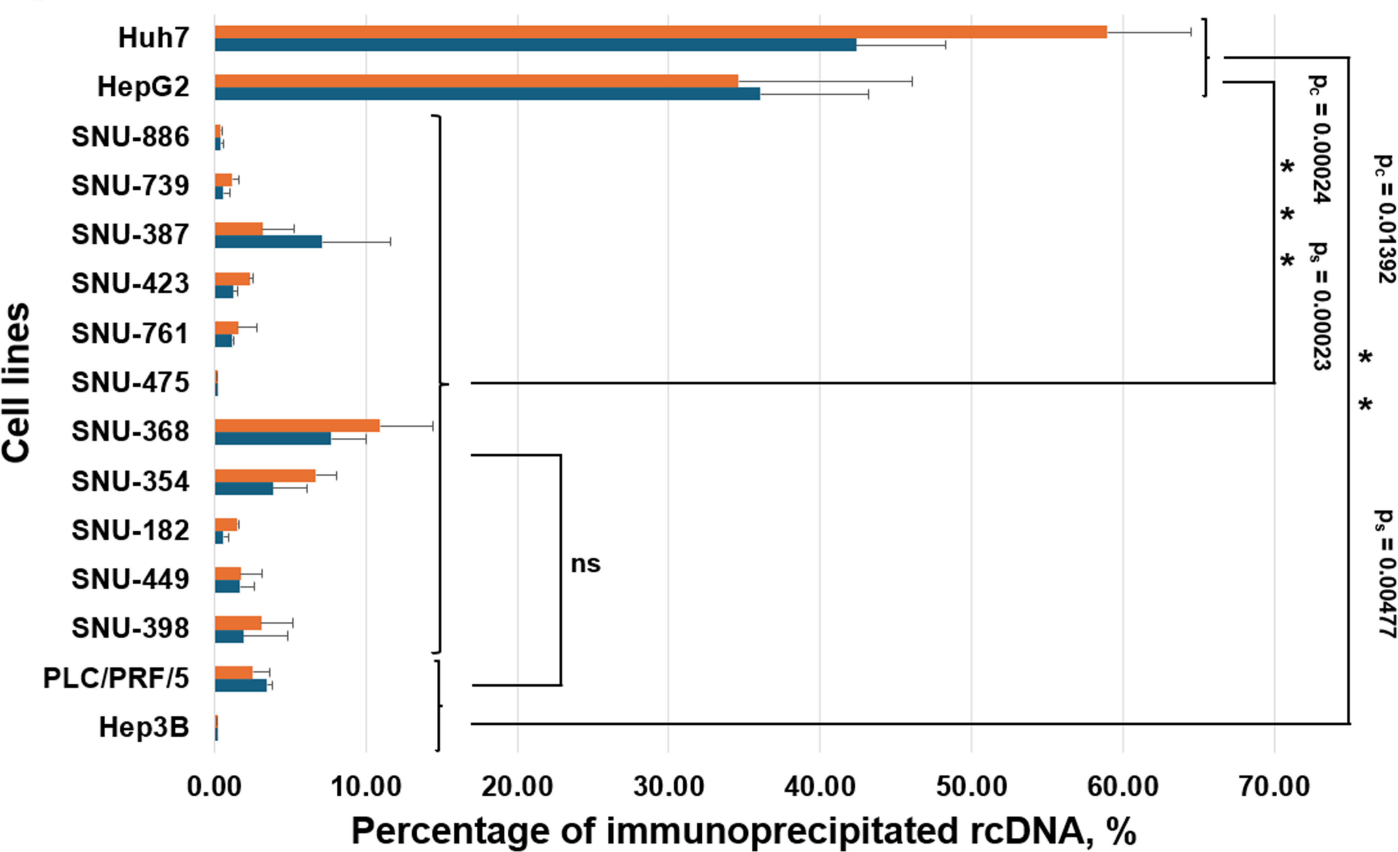
Analysis of secreted HBV DNA associated with unenveloped cores or HBsAg-enveloped virions in parental SNU cell lines using immunoprecipitation. The media were collected from parental SNU cell lines and analyzed by IP approaches either using anti-HBcAg antibodies to quantify HBV DNA associated with secreted unenveloped cores or by using anti-HBsAg antibodies to measure the secreted virion-bound HBV DNA as detailed in Materials and Methods. The same strategies were applied to the analysis of the media collected on day 8 post-transfection from control Huh7 and HepG2 cells, which were transfected with the plasmid pT-HBV1.3. As additional controls, we also analyzed the media collected from parental Hep3B and PLC/PRF/5 cells, which were developed from HBV-related HCCs and have no signs of ongoing HBV replication in them (37–41). The blue bars represent the data for the virion-associated HBV DNA, while orange bars display the data for the core-bound HBV DNA. The data (average value) are presented as the percentage of total level of secreted HBV DNA in the media. We used a pairwise Mann-Whitney *U* test and compared three sets of the cell groups (two groups of cells in each set): (i) parental SNU cell lines versus control Huh7 cells and HepG2 cells that were transfected with pT-HBV1.3; (ii) parental SNU cell lines versus two reference cell lines, PLC/PRF/5 and Hep3B; and (iii) PLC/PRF/5 and Hep3B cell lines versus control Huh7 cells and HepG2 cells that were transfected with pT-HBV1.3. As expected, the test found a statistically significant difference between parental SNU cell lines and control transfected Huh7 cells and HepG2 cells. In this case, the null hypothesis that there is no difference between those two groups can be rejected. Only control-transfected cell lines support efficient HBV replication and secrete considerable amounts of core-bound and virion-bound HBV DNA. The test did not find a statistically significant difference between parental SNU cell lines and reference Hep3B and PLC/PFR/5 cell lines, and thus, the null hypothesis in this case cannot be rejected, which was expected, because both groups of the above-mentioned cell lines are derived from HBV-related HCCs, and none of these cell lines (in both groups) still maintain any considerable levels of HBV replication, and none of them secrete any considerable levels of core-bound and virion-bound HBV DNA. Also, as expected, a significant difference was found between PLC/PRF/5 cells and Hep3B cells versus transfected Huh7 and HepG2 cells, and thus, the null hypothesis that there is no difference between these two groups can be rejected. Similarly to the comparison of the first two groups of the cell lines, only transfected Huh7 and HepG2 cells support efficient HBV genome replication and secrete considerable levels of core-bound and virion-bound HBV DNA. The results of the statistical analysis are reflected in the figure (*p*_*c*_ is *P*-value for the IP for HBcAg [analysis of secreted core-bound HBV DNA], and *p*_*s*_ is *P*-value for the IP for HBsAg [analysis of secreted virion-associated HBV DNA]).

In addition, the secreted HBV DNA associated with unenveloped (naked) cores and HBsAg-coated HBV virons was analyzed in the media collected from parental SNU cell lines and from the above-mentioned transfected Huh7 and HepG2 cell lines, which were replicating HBV genomes. The core-bound HBV DNA was assayed using the immunoprecipitation (IP) employing anti-HBcAg antibodies, and virion-associated HBV DNA was analyzed with IP using anti-HBsAg antibodies as we did previously (12). The results are shown in Fig. 3. While approximately 59% of core-bound and about 42% of virion-bound HBV DNA were found for transfected Huh7 cells, and about 35% of core-bound and 36% of virion-bound HBV DNA were observed for transfected HepG2 cells, no significant levels of core-associated or virion-associated HBV DNA were detected for the SNU cell lines (Fig. 3). As additional references, we also analyzed media from parental Hep3B and PLC/PRF/5 cells. We found no core-associated HBV DNA and no virion-associated HBV DNA for Hep3B cells, and insignificant amounts of core-bound and virion-bound HBV DNA for PLC/PRF/5 cells (Fig. 3), which was similar to the findings for the SNU cell lines. The above-described results are again consistent with absent or significantly suppressed HBV genome replication in the parental SNU cell lines.

Overall, while the analysis of the replication markers suggested the absence of HBV genome replication at least in SNU-423, SNU-368, and SNU-398 cell lines, other approaches were needed to support these initial conclusions and to further evaluate the status of HBV genome replication in the rest of the SNU cell lines.

### Coverage of selected HBV genome regions by RNA reads in parental SNU cell lines

The RNA-sequencing approach that employs the selection for polyadenylated RNA species (RNA-seq) was used next for further examination of HBV-related RNA species accumulated in parental (untransfected) SNU cell lines. First, we calculated the number of HBV-specific RNA reads for each SNU cell line, determined consensus HBV genomic sequences based on HBV-specific RNA reads, determined the sizes of HBV genomes, HBV genotype, and found close matches for full-length HBV genomes previously deposited in the NCBI database (Table S1). Since no HBV-specific RNA reads were detected for SNU-398 and SNU-423, we completed the analysis of the consensus HBV genomes only for 9 out of 11 SNU cell lines. All assembled consensus sequences for the remaining nine SNU cell lines belonged to the genotype C of HBV. Their length ranged from 3,215 to 3,378 nts. Among the reconstructed viral genomic sequences, there were no exact matches to previously reported complete HBV genomes in the NCBI database. The levels of the sequence identity of the determined consensus HBV genomes to already known HBV genomic sequences varied from 96.27%–99.32% (Table S1).

We next examined the coverage of certain HBV genome regions by RNA reads present on intracellular HBV-related RNAs, as we described previously (12). The outcomes of the analysis are presented in Table 2. The selected regions for the coverage analysis represent the unique areas of HBV genomic sequences that are expected to be present only once on rd-RNAs produced by HBV genome replication (Fig. 1 [12]). The 3,215 nucleotide (nts) HBV genome sequence GQ358158 was used as the reference viral genome (12). The area covered by the selected regions spans the positions 2,000–3,215/1–1,739 with a few minor gaps because the selected regions were adjusted to better reflect the unique sequences present on specific rd-RNA species, including pre-core/pgRNA, L mRNA, M/S mRNA, S mRNA, X mRNA and SubX mRNA (Fig. 1). This area excludes the redundant regions present on the 5′-ends and 3′-ends of the pre-core/pgRNA. The RNA reads coverage analysis did not discriminate between pre-core RNA and pgRNA (12). In addition, Table 2 also shows the area covering positions (pos.) 1,831–1,931 (as per positions in the reference HBV genome sequence GQ358158, Table 2) that is expected to be present almost exclusively on the 3′-ends of rd-RNAs, which are anticipated to bear this entire area and to be polyadenylated approximately within the region of pos. 1,929–1,953 using the HBV conventional polyadenylation signal (PAS) (12). The eight short regions spanning the area of pos. 2,000–2,800 (as per positions in the reference sequence GQ358158, Table 2) are present only on pre-core/pgRNAs of rd-RNAs, and all these regions should be covered by HBV-specific RNA reads if the intracellular HBV genome replication takes place. The absence of RNA reads coverage in any of those eight short regions argues against the presence of ongoing HBV genome replication. Given that there was a possibility of reads coverage in the 2,000–2,800 area on 5′-human-HBV-3′ RNAs that were initiated from the human promoter upstream of the integrated HBV DNA and then crossed into the integrated HBV sequence (Fig. 1), we further examined the presence of RNA reads coverage in the above-mentioned 1,831–1,931 area along with the coverage in the 2,000–2,800 region. The parental SNU cell lines, SNU-423, SNU-368, SNU-182, SNU-449, and SNU-398, have no reads in the 2,000–2,800 and 1,831–1,931 areas. As mentioned above, no HBV-specific reads were found for two cell lines—SNU-423 and SNU-398. SNU-475 has only a single read in only one region in the 2,000–2,800 area and no reads in the 1,831–1,931 region, and SNU-354 showed only one read in only one of eight regions within the 2,000–2,800 area and 32 reads in the 1,831–1,931 area. In this case, the observed 32 reads in the 1,831–1,931 region for SNU-354 are unlikely to originate from rd-RNAs, but likely from 5′-human-HBV-3′ RNA species. This observed reads coverage strongly suggests the absence of ongoing HBV genome replication in the above-mentioned seven SNU cell lines (SNU-423, SNU-368, SNU-182, SNU-449, SNU-398, SNU-475, and SNU-354). In the cell line SNU-387, we found a small number of reads only in three of eight regions that belong to the 2,000–2,800 area, and only three reads in the 1,831–1,931 region. Such a pattern again strongly suggests the absence of HBV replication in the SNU-387 cell line. In SNU-739, there were small numbers of reads in seven of eight regions in the 2,000–2,800 area (no reads in the 2,101–2,200 region), and only two reads in the 1,831–1,931 region. This reads coverage is also rather consistent with the absence of ongoing HBV replication in the SNU-739 cell line. Therefore, the above findings are consistent with the analysis of HBV replication markers in parental SNU cell lines, which suggested the absence of HBV replication in SNU-423, SNU-368, and SNU-398 cell lines (Table 1).

**TABLE 2:** Coverage by RNA reads of selected regions of the HBV genome present on HBV RNA species^*a*^

Area on reference HBV genome (GQ358158)	Area present on certain HBV mRNAs	Cell line from which total RNA was extracted											
		Huh7/HBV^*b*^ reads	SNU 886 reads	SNU 739 reads	SNU 387 reads	SNU 423 reads	SNU 761 reads	SNU 475 reads	SNU 368 reads	SNU 354 reads	SNU 182 reads	SNU 449 reads	SNU 398 reads
2,000–21,000	pc/pg	492	10	2	0	0	17	1	0	0	0	0	0
2,101–2,200	pc/pg	191	1	0	1	0	1	0	0	0	0	0	0
2,201–2,300	pc/pg	454	4	1	1	0	8	0	0	0	0	0	0
2,301–2,400	pc/pg	746	13	5	4	0	8	0	0	0	0	0	0
2,401–2,500	pc/pg	409	32	3	0	0	5	0	0	0	0	0	0
2,501–2,600	pc/pg	452	12	2	0	0	1	0	0	0	0	0	0
2,601–2,700	pc/pg	483	20	4	0	0	4	0	0	1	0	0	0
2,701–2,800	pc/pg	599	16	1	0	0	6	0	0	0	0	0	0
2,801–2,900	pc/pg, L	889	32	2	2	0	49	0	1	28	0	0	0
2,901–3,180	pc/pg, L	1,862	100	5	3	0	170	2	2	44	0	0	0
3,183–3,215	pc/pg, L, M/S	207	21	2	0	0	43	0	4	17	0	0	0
1–15	pc/pg, L, M/S	197	2	6	0	0	63	0	7	18	0	0	0
25-200	pc/pg, L, M/S, S	2,084	101	32	3	0	1,078	0	41	147	0	0	0
201-400	pc/pg, L, M/S, S	3,368	150	52	2	0	1,631	0	57	254	1	1	0
401-600	pc/pg, L, M/S, S	3,026	63	23	1	0	794	0	27	120	1	1	0
601-800	pc/pg, L, M/S, S	1,343	51	13	0	0	521	0	13	56	2	5	0
801-1,000	pc/pg, L, M/S, S	1,503	43	20	12	0	634	0	19	74	1	7	0
1,001-1,200	pc/pg, L, M/S, S	3,313	93	41	8	0	1,201	0	40	146	10	11	0
1,221-1,350	pc/pg, L, M/S, S, X	2,711	76	43	6	0	1,578	2	27	193	27	33	0
1,351-1,500	pc/pg, L, M/S, S, X	5,451	153	64	5	0	2,457	2	68	280	36	78	0
1,531-1,630	pc/pg, L, M/S, S, X, SubX	3,903	134	36	1	0	1,025	0	39	75	20	27	0
1,631-1,739	pc/pg, L, M/S, S, X, SubX	2,721	93	27	4	0	689	1	30	68	25	14	0
1,831-1,931	All rd-RNAs^*c*^	1,986	51	2	3	0	180	0	0	32	0	0	0

^*a*^ The coverage of selected regions of the HBV genome that could be present on HBV RNA species was deduced from the analysis of RNA-seq data. The RNA samples isolated from parental SNU cell lines and Huh7 cells transfected with the plasmid pT-HBV1.3 were examined. The regions selected for the analysis are present only once on the indicated types of rd-RNAs: pc/pg, pre-core/pre-genomic RNAs; L, L mRNA; M/S, M/S mRNA; S, S mRNA; X, X mRNA; and SubX, SubX mRNA. All these HBV mRNAs are described in the text (Fig. 1). Importantly, the regions covering the area of HBV positions 2,000–2,800 are only present on pgRNA and pre-core RNA produced by HBV genome replication. The positions of selected regions are indicated for the reference HBV genome (GQ358158) on the left-hand side. For the HBV variant found in a particular SNU cell line, the numbering could differ, while a selected region will represent the same area with a considerably homologous, if not identical, viral sequence. Note that no HBV-specific RNA reads were recovered for SNU-423 and SNU-398.

^*b*^ RNA isolated from Huh7 cells transfected with the construct pT-HBV1.3 and replicating HBV genome (also shown as Huh7/HBV) served as a control. It was used as the control sample for comparison with the material isolated from parental (untransfected) SNU cell lines.

^*c*^ The area spanning HBV positions 1,831–1,931 is expected to be present almost exclusively at the 3′-ends of RNAs produced by HBV genome replication and polyadenylated using HBV conventional polyadenylation signal (i.e., rd-RNAs) (Fig. 1 [9, 12]).

All the above observations were in a strong contrast with the HBV-specific RNA reads coverage in transfected Huh7 cells replicating the HBV genome, for which extensive numbers of RNA reads were found in all regions selected for the analysis (Table 2). Furthermore, the analysis of the RNA reads coverage described above also strongly suggests that the measured RNA numbers by RT-qPCRs aimed to detect total HBV RNA, rd-RNAs, and pre-core/pgRNAs displayed in Table 1 for SNU-423, SNU-475, SNU-368, SNU-354, SNU-182, SNU-449, SNU-398, SNU-387, and SNU-739 reflect the presence of diverse 5′-human-HBV-3′ and 5′-HBV-human-3′ RNA species transcribed from integrated HBV DNA in the absence of ongoing HBV genome replication (Fig. 1). As described above, we also observed either undetermined or very small levels for the intracellular core-associated HBV DNA in all these cell lines, and undetermined or very small numbers for cccDNA in six out of nine of these cell lines (with the exception of SNU-739, SNU-475, and SNU-398) (Table 1). The cases, when core-associated DNA or cccDNA were measurable, (i) very likely reflect the known situation that none of the specific DNA isolation procedures used can completely prevent the presence of the residual amounts of apparently fragmented chromosomal DNA bearing integrated HBV DNA in the final DNA preparations (42–45), which were amplified by the qPCR assays used, and (ii) do not actually reflect ongoing HBV genome replication.

The data looked somewhat different for the remaining SNU-886 and SNU-761 cell lines. They both displayed a number of HBV-specific reads in all regions within the 2,000–2,800 area and also had 51 and 180 reads in the 1,831–1,931 region, respectively (Table 2). This suggests that there could be some residual HBV genome replication still present in these two SNU cell lines, and that the levels of measured total HBV RNA, rd-RNAs, and pre-core/pgRNA, as well as core-associated DNA and cccDNA (Table 1), could reflect in part the RNA and DNA species produced by HBV genome replication, and, in part, integrant-transcribed RNAs and chromosomal fragments bearing integrated HBV DNA, respectively.

The RNA reads coverage in the downstream regions that belong to the area of pos. 2,801–3,215/1–1,739 is consistent with the presence of the L mRNA and M/S mRNA sequences for SNU-886, SNU-739, SNU-761, SNU-368, and SNU-354. The presence of S mRNA sequences was indicated for SNU-886, SNU-739, SNU-761, SNU-368, SNU-354, and SNU-387 (but not in all S regions for the last cell line). The presence of X mRNA sequences was indicated for SNU-886, SNU-739, SNU-761, SNU-368, SNU-354, SNU-449, SNU-387, SNU-182, and SNU-475 (but not in all X regions for the last cell line). The presence of SubX mRNA sequences was indicated for SNU-886, SNU-739, SNU-761, SNU-368, SNU-354, SNU-182, SNU-449, and SNU-387. All HBV-specific RNA reads found in the above-mentioned regions of HBV sequences, however, can (i) represent RNAs transcribed from integrated DNA independently of HBV genome replication using the viral envelope or X or SubX promoters that could remain intact and functional in the context of HBV DNA integrants, or (ii) alternatively be produced by HBV replication (if HBV replication still takes place in a particular cell line).

### Polyadenylation sites on HBV-related RNAs within HBV sequences

Using the bioinformatic approach described by us previously (12), we analyzed polyadenylation sites within the HBV sequence, focusing on RNA reads generated by RNA-seq that bear stretches of As at their very 3′-ends downstream of HBV sequences, which do not belong to the HBV sequence. We considered only the reads bearing at least three As at their 3′-ends. The results of the analysis are summarized in Table S2. First, no poly(A) addition sites within the HBV sequence were detected for SNU-423, SNU-475, SNU-368, SNU-182, and SNU-398. In addition, no poly(A) addition sites within the area of pos. 1,929–1,953, which was reported by us as the area reflecting most polyadenylation sites corresponding to the use of the conventional HBV PAS (TATAAA spanning the pos. 1,916–1,921 in the reference HBV genome sequence GQ358158) (12), were found for SNU-449 (Table S2). These findings were consistent with the above-mentioned conclusions of the absence of ongoing HBV genome replication in SNU-423, SNU-475, SNU-368, SNU-182, SNU-449, and SNU-398 cell lines. Furthermore, for SNU-387 and SNU-739, we detected only a single read per cell line for the polyadenylation sites at pos. 1,936 or 1,939, respectively, which resided in the above-mentioned area of pos. 1,929–1,953, which argues against the presence of HBV genome replication in these two cell lines as well. It needs to be remembered that HBV RNAs bearing polyadenylation sites in the HBV sequence, including the area 1,929–1,953, may be originated not only from HBV replication but also represent 5′-human-HBV-3′ RNAs transcribed from integrated HBV DNA independently of HBV replication, which were polyadenylated within integrated HBV sequence (Fig. 1). Eleven reads corresponding to polyadenylation in the 1,929–1,953 area were detected for SNU-354 (corresponding to the pos. 1,935, 1,936, 1,941, 1,944, and 1,947 in the reference HBV sequence GQ358158). Since the cell line SNU-354 was evaluated above as the one not bearing ongoing HBV genome replication, these polyadenylation sites should correspond to the HBV integrant-transcribed RNAs. It should also be noted that, depending on the boundaries (left-hand side and right-hand side positions) of the integrated HBV DNA, some HBV RNAs transcribed from HBV integrants could be initiated from the HBV promoter and contain only HBV sequences. These RNAs may acquire polyadenylation within the area 1,929–1,953 if the integrated HBV DNA extends far enough beyond pos. ~1,929 ([7], Fig. 1). This situation, however, is expected to be the case only for a minority of HBV integrations. For SNU-886 and SNU-761, we detected 4 and 87 polyadenylation sites, respectively, within the area 1,929–1,953 (Table S2), which is consistent with possible residual HBV replication still occurring in these cell lines, as it was evaluated in the previous sections of the paper.

We noted that there were some differences between the read number for the coverage of the area 1,831–1,931 (Table 2) and the number of reads reflecting the polyadenylation sites within the area 1,929–1,953 (Table S2) that bear some sequences of the 1,831–1,931 area, which reflect the differences in the algorithms used to generate the data for the two above-mentioned tables and the differences in the boundaries of the two above-mentioned regions. These differences, however, did not affect our evaluation of the data.

As the cryptic HBV PAS (CATAAA) is located at pos. 1,788–1,793 (per reference HBV genome GQ358158 numbering) and given that polyadenylation frequently occurs within about 10–35 nucleotides downstream of the PAS (47), it seems that SNU-886, SNU-761, and SNU-449 may contain a few RNAs polyadenylated via the cryptic HBV PAS at positions equivalent to pos. 1,811 in the reference sequence. There were also a number of other infrequent poly(A) addition sites found at several other positions for SNU cell lines, with the exception of SNU-449, for which the two reads reflecting polyadenylation at pos. 1,811 were the only detected polyadenylation sites (Table S2).

Table 2S also includes the data for the Huh7 cells transfected with pT-HBV1.3 and replicating HBV genome as the reference data set. In Huh7 cells replicating HBV genome, the most poly(A) addition sites were as expected within the area 1,929–1,953, thus representing most of the RNAs produced by the viral replication and polyadenylated via conventional HBV PAS (i.e., rd-RNAs) (12). Other frequent poly(A) sites for transfected Huh7 cells were at pos. 870, 929, and 2,924 (reference HBV genome GQ358158 numbering). There were also quite a few infrequent poly(A) sites mapped throughout the HBV genome. Among them were the sites in the area from pos. ~1,800 to ~1,827 that could represent polyadenylation via cryptic HBV PAS. The biological significance of other detected poly(A) sites, which were mostly infrequent and found in different cell lines and cannot be attributed to the polyadenylation mediated by HBV conventional or cryptic PAS (Table S2), is not immediately apparent and could be investigated further in follow-up studies.

### Analysis of intracellular RNAs transcribed from integrated HBV DNA

Using RNA-seq data, we analyzed 5′-human-HBV-3′ RNAs and 5′-HBV-human-3′ RNAs (i.e., integrant-derived RNAs or id-RNAs [9]) (Fig. 1) as previously described (12). These two types of HBV integrant-transcribed RNAs are generated from integrated HBV DNA independently of HBV genome replication. We found such RNA species in parental cell lines SNU-886, SNU-739, SNU-387, SNU-761, SNU-368, SNU-354, SNU-182, and SNU-449. We also analyzed integrant-transcribed RNAs in Huh7 cells replicating the HBV genome (post-transfection with pT-HBV1.3) that served as the control. The results of the analysis are presented in Table 3.

**TABLE 3:** Integrant-transcribed HBV RNAs in parental SNU cell lines and in transfected Huh7 cells replicating HBV genome^*a*^

Sample	5′-element^*b*^	5′ -chr^*c*^	5′-pos^*d*^ (actual )^*l*^	5′-pos^*e*^	3′-element^*f*^	3′ -chr^*g*^	3′-pos^*h*^ (actual)^*l*^	3′-pos^*i*^ (GQ358158)	RNA read count	Gene region^*j*^	Human gene^*k*^
				(GQ358158)							
SNU-886	Human	1	116,404,494		HBV		2,442	2,279	1	ncRNA_intronic	ATP1A1-AS1
	Human	9	42,454,642		HBV		2,014	1,851	2	Intergenic	FAM74A7/LOC103908605
	Human	11	69,298,648		HBV		1,994	1,831	17	Downstream	MYEOV
	Human	17	19,061,982		HBV		2,778	2,615	1	ncRNA_intronic	SNORD3B-1, SNORD3B-2
	HBV		1,990	1,827	Human	12	122,349,058		1	Intronic	CLIP1
	HBV		179	179	Human	1	240,143,376		1	Intronic	FMN2
	HBV		2,113	1,950	Human	9	42,456,649		20	Intergenic	FAM74A7/LOC103908605
SNU-739	Human	13	27,620,509		HBV		2,299	2,299	3	5UTR^*m*^	LNX2
	HBV		1,824	1,824	Human	4	25,185,899		25	ncRNA_intronic	SEPSECS-AS1
	HBV		2,447	2,447	Human	13	28,037,288		5	Exonic	FLT3
	HBV		2,472	2,472	Human	13	28,036,042		1	Exonic	FLT3
SNU-387	Human	17	47,489,728		HBV		829	829	15	ncRNA_intronic	MRPL45P2
	HBV		1,364	1,364	Human	16	65,090,590		1	Intronic	CDH11
	HBV		1,374	1,374	Human	16	65,090,590		1	Intronic	CDH11
SNU-761	Human	2	32,227,423		HBV		1,928	1,840	2	Intronic	NLRC4
	Human	2	71,682,627		HBV		402	402	18	Exonic	DYSF
	Human	ND	115,064		HBV		263	263	1	Intergenic	Unidentified/unidentified
	Human	19	6,696,487		HBV		983	983	1	Intronic	C3
	HBV		1,776	1,745	Human	2	117,941,254		2	Exonic	CCDC93
	HBV		1,668	1,644^*o*^	Human	11	81,934,233		1	ncRNA_intronic	MIR4300HG
	HBV		1,734	1,703	Human	5	1,297,698		47	Upstream	TERT
	HBV		1,461	1,461	Human	1	180,197,370		1	3UTR^*n*^	QSOX1
	HBV		420	420	Human	2	71,686,446		18	Intronic	DYSF
	HBV		1,631	1,631	Human	13	30,460,103		1	3UTR^*n*^	HMGB1
	HBV		1,856	1,810^*o*^	Human	9	75,299,907		62	Intergenic	OSTF1/PCSK5
	HBV		1,946	1,858	Human	2	32,227,443		2	Intronic	NLRC4
	HBV		1,670	1,644^*o*^	Human	11	81,934,234		25	ncRNA_intronic	MIR4300HG
	HBV		1,842	1,810^*o*^	Human	5	1,192,299		4	Upstream	SLC6A19
SNU-368	Human	3	88,475,823		HBV		1,733	1,733	1	Downstream	CSNKA2IP
	HBV		1,105	1,105	Human	2	42,304,490		1	Exonic	EML4
	HBV		1,784	1,784	Human	3	88,475,758		39	Downstream	CSNKA2IP
SNU-354	Human	ND	117,380		HBV		1,705	1,705	2	Intergenic	Unidentified/unidentified
SNU-182	HBV		1,813	1,813	Human	1	175,455,936		7	Intronic	TNR
SNU-449	Human	5	1,281,057		HBV		1,451	1,451	3	Intronic	TERT
	Human	12	105,195,399		HBV		485	485	2	Intronic	APPL2
	HBV		1,362	1,362	Human	1	203,752,441		1	Downstream	ATP2B4
	HBV		1,803	1,803	Human	10	37,404,639		1	Intergenic	LINC00993/MTRNR2L7
	HBV		1,810	1,810	Human	12	105,191,350		2	Intronic	APPL2
Huh7/HBV	Human	3	119,436,947		HBV		1,398	1,398	1	Exonic	TMEM39A
	Human	9	36,676,098		HBV		419	419	2	Intronic	MELK
	Human	14	102,029,955		HBV		264	264	1	Intronic	DYNC1H1
	Human	3	133,754,650		HBV		646	646	1	Exonic	TF
	Human	X	141,118,166		HBV		2,838	2,838	1	Upstream	RNU6-1,RNU6-2, RNU6-7, RNU6-8, RNU6-9
	Human	15	55,204,117		HBV		160	160	1	3UTR^*n*^	RAB27A
	Human	12	57,212,818		HBV		1,600	1,600	1	3UTR^*n*^	LRP1
	Human	2	174,677,770		HBV		2,018	2,018	2	Intronic	WIPF1
	Human	ND	110,419		HBV		1,999	1,999	2	Intergenic	Unidentified/unidentified
	Human	17	76,723,925		HBV		1,764	1,764	2	Exonic	JMJD6
	Human	ND	110,500		HBV		2,620	2,620	1	Intergenic	Unidentified/unidentified
	Human	1	8,361,354		HBV		1,204	1,204	2	Exonic	RERE
	Human	ND	110,500		HBV		1,981	1,981	1	Intergenic	Unidentified/unidentified
	Human	14	103,465,804		HBV		1,130	1,130	2	Intronic	MARK3
	Human	2	71,682,621		HBV		396	396	4	Exonic	DYSF
	Human	11	70,436,427		HBV		2,120	2,120	1	3UTR^*n*^	CTTN
	HBV		328	328	Human	19	3,977,964		1	Exonic	EEF2
	HBV		1,891	1,891	Human	6	89,316,357		1	Upstream	GABRR2
	HBV		1,825	1,825	Human	20	19,497,770		2	Intronic	SLC24A3
	HBV		1,783	1,783	Human	17	76,721,926		3	Exonic	JMJD6
	HBV		1,885	1,885	Human	8	134,130,711		1	Intergenic	LOC101927822/ZFAT
	HBV		1,819	1,819	Human	20	43,141,807		6	Intronic	PTPRT
	HBV		1,651	1,651	Human	19	8,833,432		2	Upstream	MBD3L1, ZNF558
	HBV		437	437	Human	9	36,676,118		2	Intronic	MELK
	HBV		420	420	Human	2	71,682,647		3	Exonic	DYSF

^*a*^ Table includes two types of integrant-transcribed HBV RNAs, 5′-human-HBV-3′ RNAs (shown as the top set for all cell lines [one exception here was the SNU-182 cell line, in which 5′-human-HBV-3′ RNAs were not found]), and 5′-HBV-human-3′ RNAs (id-RNAs) (shown as the bottom set of integrant-transcribed RNAs). Huh7/HBV represents Huh7 cells transfected with pT-HBV1.3 and replicating HBV genome. It was used as the control (the cells were harvested for the analysis on day 8 post-transfection).

^*b*^ 5′-element refers to human or viral 5′-part origin, thus reflecting 5′-human-HBV-3′ RNAs or 5′-HBV-human-3′-RNAs.

^*c*^ 5'-chr refers to human chromosome bearing integrated HBV DNA producing 5′-human-HBV-3′ RNAs. ND indicates the cases when the chromosome bearing HBV DNA integrant could not be identified, because HBV-containing human sequence was not yet assigned to a specific human chromosome.

^*d*^ 5′-pos reflects the position number in the human or viral 5′-part sequence of integrant-transcribed RNA at the viral/host junction site.

^*e*^ This 5′-pos (GQ358158) refers to the HBV position at the viral/host junction site for 5′-HBV-human-3′ RNA determined in the HBV genome reference sequence GQ358158.

^*f*^ 3′-element refers to human or viral 3′-part origin, thus reflecting 5′-human-HBV-3′ RNAs or 5′-HBV-human-3′-RNAs.

^*g*^ 3′-chr refers to human chromosome bearing integrated HBV DNA producing 5′-HBV-human-3′ RNAs (id-RNAs).

^*h*^ 3′-pos reflects the position number in human or viral 3′-part sequence of integrant-transcribed RNA at the virus/host junction site.

^*i*^ This 3′-pos (GQ358158) refers to the HBV position at the virus/host junction site for 5′-human-HBV-3′ RNA determined in the HBV genome reference sequence GQ358158.

^*j*^ Gene region reflects the location of the HBV integration relative to human genes (i.e., intronic, exonic, intergenic, downstream, etc.).

^*k*^ Human genes harboring integrated HBV DNA are shown as commonly used abbreviations. For some cases with intergenic location of HBV integrant, integrated HBV DNA was surrounded by coding sequences of human genes that were not characterized and annotated yet, which was marked as unidentified/unidentified in the column for the human gene serving as a recipient of HBV DNA integrant.

^*l*^ Actual refers to the HBV position in actual viral genome variant found in a particular parental SNU cell line (or in transfected Huh7 cells) at the virus/host junction (5′-pos or 3′-pos depending on the type of integrant-transcribed RNA).

^*m*^ 5UTR refers to 5′-UTR region of a human gene bearing HBV DNA integrant.

^*n*^ 3UTR refers to 3′-UTR region of a human gene bearing HBV DNA integrant.

^*o*^ This mark means that there was no exact match to the nucleotide in the HBV genome reference sequence GQ358158 for the nucleotide in the actual HBV variant sequence, and closest nt in the reference sequence was given as the reference in the table with ”o” mark instead.

**Fig 4:**
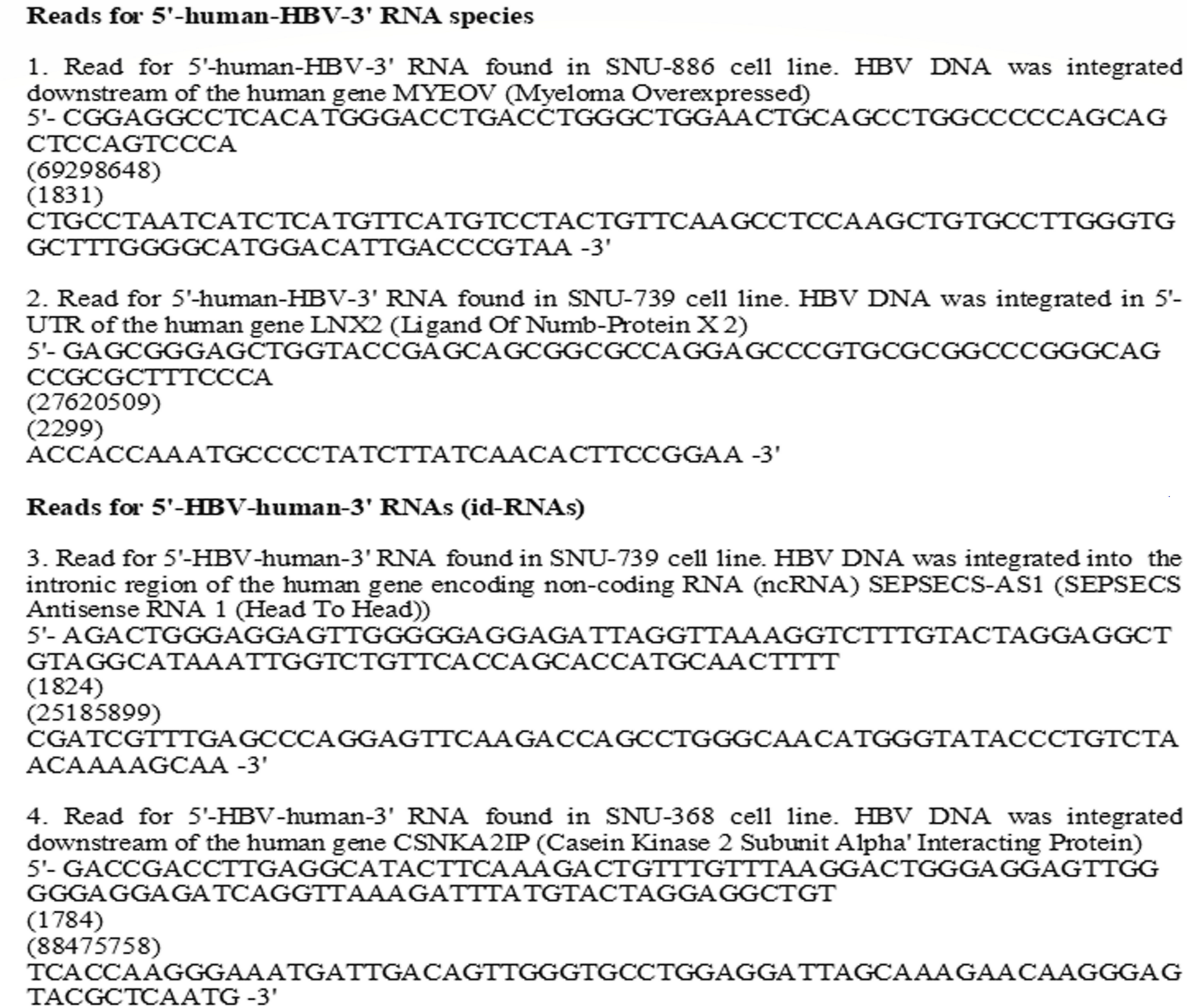
Examples of RNA reads representing integrant-transcribed HBV RNAs found in SNU cell lines. Shown are the examples of RNA reads representing two 5′-human-HBV-3′ RNAs and two 5′-HBV-human-3′ RNAs (id-RNAs) found in several SNU cell lines using RNA-seq based on poly(A) selection. For each RNA, shown are 5′- and 3′- sequences of the hybrid HBV-human integrant-transcribed RNAs and the positions for the viral (in the coordinates of the reference HBV genome GQ358158) and human sequences at the virus/host junction sites (in parentheses). In addition, human genes bearing corresponding HBV DNA integrants are indicated.

Both 5′-human-HBV-3′ RNAs and id-RNAs were found in SNU-886, SNU-739, SNU-387, SNU-761, SNU-368, and SNU-449 cell lines and also in the control-transfected Huh7 cells (Huh7/HBV). The examples of the RNA reads corresponding to either 5′-human-HBV-3′ RNAs or to 5′-HBV-human-3′ RNA species are provided in Fig. 4. For SNU-354, only one type of 5′-human-HBV-3′ RNAs was found. No id-RNAs were detected for this cell line. For SNU-182, we only detected one type of id-RNAs and no 5'-human-HBV-3′ RNAs.

SNU-886, SNU-739, SNU-387, SNU-761, SNU-368, and SNU-449 bear several HBV DNA integrants producing integrant-transcribed HBV RNAs. Thus, for example, SNU-886 had four types of 5′-human-HBV-3′ RNAs and three types of 5′-HBV-human-3′ RNAs, while SNU-739 had one type of 5′-human-HBV-3′ RNA and three types of id-RNAs. For Huh7/HBV cells, several RNA-generating HBV DNA integrants were noted as well (Table 3). The highest intracellular accumulation of integrant-transcribed HBV RNAs was found in SNU-761 cells. For the SNU cell lines, the number of transcripts for a single individual type of integrant-transcribed RNAs ranged from 1 to 62 copies of a unique RNA transcript/sample. For control Huh7 cells replicating the HBV genome, the number of reads for a unique individual type of integrant-transcribed RNAs ranged from 1 to 6.

It needs to be noted that in four of seven cases when id-RNAs were found, the number of id-RNAs reads/transcripts exceeded the number of reads potentially corresponding to rd-RNAs (SNU-739, SNU-368, SNU-182, SNU-449). Thus, for example, SNU-739 had 2 reads corresponding to rd-RNAs (Table 2), 3 reads of 5′-human-HBV-3′ RNAs, and 31 reads of id-RNAs (Table 3), while SNU-368 had 0 reads corresponding to rd-RNAs (Table 2), and 1 read corresponding to 5′-human-HBV-3′ RNAs and 40 reads of id-RNAs (Table 3). In addition, among the remaining three cell lines, the number of id-RNAs was considerable for SNU-886 and SNU-761 compared to the numbers of rd-RNAs (Tables 2 and 3).

For SNU cell lines, the RNA-generating HBV DNA integrants were located on chromosomes 1, 2, 3, 4, 5, 9, 10, 11, 12, 13, 16, 17, and 19. For Huh7/HBV cells, the RNA-producing HBV DNA integrants were located on the chromosomes 1, 2, 3, 6, 8, 9, 11, 12, 14, 15, 17, 19, 20, and X. For both SNU cell lines and Huh7/HBV cells, the majority of RNA-generating HBV DNA integrants were not located in the coding sequences (exonic regions). For SNU cell lines, they were predominantly found in intronic (including intronic regions for non-coding RNAs) and intergenic regions, in 5′-UTRs and 3′-UTRs (untranslated regions), upstream and downstream of genes. For Huh7/HBV cells, they were mostly located in intronic and intergenic areas, in 3′-UTRs and upstream of certain genes (Table 3). A similar tendency was noted by us previously during the analysis of integrant-transcribed HBV RNAs in matching liver/HCC tissues harvested from untreated individuals chronically infected with HBV (9).

While the variety of human genes bearing HBV DNA integrants in SNU cell lines and in Huh7/HBV cells reflects a random mode of HBV DNA integration (31), in SNU-761 we found one integration site upstream of telomerase reverse transcriptase (TERT), a previously reported gene that is considered to be one of the recurrent sites for HBV DNA integration (48–53). We also found one integration into the intronic region of the TERT gene in SNU-449 (Table 3). In addition, we noted several genomic locations bearing more than one HBV integration site producing HBV-related RNA species. Thus, for example, in SNU-886, it is the intergenic region between FAM74A7 (family with sequence similarity 74 member A7) and LOC103908605 (uncharacterized LOC103908605 long non-coding RNA [from RefSeq NR_126050]) genes, containing two HBV integrants, while SNU-739 bears two HBV integrants in the exonic region of FLT3 (Fms Related Receptor Tyrosine Kinase class III that regulates hematopoiesis), and SNU-387 contains two integrants in the intronic region of CDH11 (Cadherin 11) gene (Table 3). For Huh7/HBV cells, we also found two integrations per genomic location for MELK (Maternal Embryonic Leucine Zipper Kinase), DYSF (dysferlin), and JMJD6 (Jumonji Domain Containing 6, Arginine Demethylase and Lysine Hydroxylase) (Table 3).

Table 3 also indicates the locations of the left-hand side and right-hand side of the HBV DNA integrants based on the mapped HBV positions at the virus-host junction sites in SNU cell lines. It became apparent that a number of 5′-human-HBV-3′ RNAs were likely produced from integrated double-stranded linear HBV DNA genomes that were missing HBV sequences from the left-hand side (Fig. 1). Such cases, for example, include the HBV positions (in GQ358158 coordinates) 2,615 and 2,279 (SNU-886), 2,299 (SNU-739), and 1,999, 2,018, 2,120, 2,620, and 2,838 (in transfected Huh7 cells) (Table 3). In some other cases, DSLs that served as the templates for the transcription of 5′-human-HBV-3′ RNAs were not truncated or lost just a bit of sequence at the left-hand side. For example, this was the case for the left-hand side pos. 1,764 (Huh7/HBV); 1,851, 1,831 (SNU-886); 1,840 (SNU-761); 1,733 (SNU-368); and 1,705 (SNU-354) (Table 3).

For the right-hand side, it seems that quite a few integrated DSLs were not truncated there or lost just small sequence areas (for example, the positions 1,827, 1,824, 1,745, 1,810^o^ [when there was no exact match in the HBV reference genome to the actual nucleotide in the HBV variant found in a particular SNU cell line, the nearest nucleotide in the GQ358158 reference sequence was indicated with an “o” mark instead of the exact match], 1,858, 1,784, 1,813, 1,803, and 1,810 for SNU cell lines [Table 3]). There were also cases that were consistent with the loss of a considerable portion of right-hand side sequences (for example, the positions 179, 1,364, 1,374, 1,461, 420, 1,105, and 1,362 for SNU cell lines [Table 3]). The latter may also represent the integration events for the HBV DNA fragment smaller than the complete DSL.

In addition, there were also integrants with left-hand side positions apparently extended further into upstream HBV sequences (such as pos. 1,451 in SNU-449, and 1,398, 1,600, 1,204, etc., in Huh7/HBV) and integrants with right-hand side positions extended further into downstream HBV sequences compared to the expected corresponding positions of the complete DSL (such as pos. 1,950 in SNU-886, and 2,447 and 2,472 in SNU-739) (Fig. 1; Table 3).

Interestingly, it became apparent that based on the positions of the right-hand side of HBV integrants, several SNU cell lines could potentially be used as the model systems for detailed analysis of the properties of 5′-HBV-human-3' RNAs (id-RNAs) encoding HBV envelope proteins (ORF for which ends at pos. 835 of the reference sequence [34]). The additional assumption here was that the integrants of interest were full-length DSLs with functional envelope promoters. This applies to SNU-886, SNU-739, SNU-387, SNU-761, SNU-368, SNU-182, and SNU-449, which all bear the integrants with the right-hand side positions downstream of HBV pos. 835 (Table 3). However, the analysis of the HBV RNA reads coverage (Table 2) only supports the possible production of id-RNAs encoding HBsAg for the SNU-886, SNU-761, SNU-739, and SNU-368 cell lines. The additional benefit for SNU-739 and SNU-368 cell lines is that they were evaluated as the ones without ongoing HBV genome replication, which could allow to have pure experimental systems to study HBsAg-encoding id-RNAs in the absence of concomitant HBV replication. Similarly, Huh7/HBV cells could also be used as controls for the production of HBsAg-encoding id-RNAs as they also bear the integrants with the right-hand sides extended beyond HBV pos. 835 (Table 3). However, in the case of Huh7/HBV cells, there will be a concomitant HBV replication.

### Spliced HBV RNAs

Table 4 summarizes the analysis of spliced HBV RNA species found using RNA-seq in parental SNU cell lines and in control Huh7 cells transfected with pT-HBV1.3 and replicating HBV genome. No spliced HBV RNAs were detected in SNU-449, SNU-182, and SNU-368 cell lines. As mentioned above, SNU-423 and SNU-398 cell lines had no HBV-specific RNA reads, and SNU-475 had only a total of 73 HBV-specific RNA reads (Table S1), and thus, there were no sufficient numbers of reads to analyze spliced HBV RNAs for these three cell lines. The rest of SNU cell lines, SNU-886, SNU-739, SNU-387, SNU-761, and SNU-354, displayed between two and seven spliced HBV RNA variants each. For transfected Huh7 cells that served as a control, we identified 23 different variants of spliced HBV RNAs. Both canonical (CT/AC and GT/AG) and non-canonical intron motifs were found for detected spliced RNAs. The size of the splicing-generated deletions ranged from 30 to 2,593 nucleotides (Table 4).

**TABLE 4:** Spliced HBV RNAs in parental SNU cell lines and in transfected Huh7 cells replicating HBV genome^*g*^

Sample	Intron motif^*a*^	Known Sp variant^*b*^	Found also in^*c*^	Found in serum^*d*^	Splice start (GQ358158)^*e*^	Splice end (GQ358158)^*e*^	Splice start (actual)^*e*^	Splice end (actual)^*e*^	Length (nts)	Unique splice reads	Percentage^*f*^
SNU-886	CT/AC				2,110	2,755	2,273	2,918	646	1	1.65
	CT/AC		387		2,110	342	2,273	342	1,448	8	5.59
	GT/AG	Sp1	761, Ctr	Y	2,448	488	2,611	488	1,256	2	2.2
	GT/AG				2,472	2,934	2,635	3,097	463	1	1.04
	CT/AC				2,605	338	2,768	338	949	2	1.37
	CT/AC				144	342	144	342	199	2	1.08
	CT/AC				1,595	1,694	1,595	1,706	112	148	59.08
SNU-739	Non-canonical				22	54	22	54	33	6	13.04
	CT/AC				144	338	144	338	195	2	3.39
SNU-387	CT/AC				2,110	338	2,110	338	1,444	3	13.95
	CT/AC		886		2,110	342	2,110	342	1,448	2	9.3
	Non-canonical				8	55	8	55	48	12	66.67
SNU-761	CT/AC				1,810	1,828	1,860	1,916	57	176	89.11
	GT/AG				2,156	2,251	2,244	2,339	96	17	94.44
	GT/AG	Sp1	886, Ctr	Y	2,448	488	2,536	488	1,256	2	0.41
	GT/AG				2,452	2,718	2,540	2,806	267	1	7.41
	CT/AC		Ctr		124	324	124	324	201	1	0.07
	GT/AG				1,644	1,646	1,647	1,677	31	578	58.62
SNU-354	GT/AG				1,801	1,847	1,802	1,848	47	26	96.3
	GT/AG	pSP12	Ctr	Y	3,019	281	3,020	281	478	2	1.09
	GT/AG	Sp14	Ctr	Y	3,019	488	3,020	488	685	2	2.04
	GT/AG	Sp21	Ctr	Y	459	1,304	459	1,304	846	1	0.45
Huh7/HBV	CT/AC				1,820	2,676	1,820	2,676	857	2	0.14
(Ctr)^*c*^	CT/AC				1,831	2,325	1,831	2,325	495	1	0.06
	CT/AC				1,892	1,271	1,892	1,271	2,593	2	0.07
	CT/AC				1,935	921	1,935	921	2,200	2	0.11
	CT/AC			Y	1,935	928	1,935	928	2,207	117	6.52
	CT/AC				1,935	2,561	1,935	2,561	627	110	6.6
	CT/AC				1,948	921	1,948	921	2,187	1	0.06
	GT/AG	Sp13		Y	2,448	2,934	2,448	2,932	485	44	2.09
	GT/AG	Sp9		Y	2,448	281	2,448	281	1,047	18	0.62
	GT/AG	Sp1	886, 761	Y	2,448	488	2,448	488	1,254	344	13.04
	GT/AG	Sp11			2,472	281	2,472	281	1,023	4	0.14
	GT/AG	Sp6		Y	2,472	488	2,472	488	1,230	13	0.49
	Non-canonical				2,864	2,901	2,864	2,894	31	1,359	58.79
	Non-canonical				2,884	2,913	2,880	2,911	32	4	0.17
	GT/AG				3,019	3,115	3,017	3,113	97	6	0.25
	GT/AG	pSP12	354	Y	3,019	281	3,017	281	478	42	1.27
	GT/AG	Sp14	354	Y	3,019	488	3,017	488	685	98	3.23
	CT/AC		761		124	324	124	324	201	13	0.33
	GT/AG				459	488	459	488	30	2	0.05
	GT/AG				459	1,086	459	1,086	628	1	0.03
	GT/AG	Sp21	354	Y	459	1,304	459	1,304	846	1	0.03
	GT/AG	pSP10(2/2)^*b*^			459	1,307	459	1,307	849	4	0.11
Huh7/HBV (Ctr)^*c*^(cont.)	GT/AG	Sp22			459	1,384	459	1,384	926	3	0.08

^*a*^ The 5′- and 3′- dinucleotides in the intron motif have been used during the identification of HBV RNA sequences that were spliced. The approach for identifying spliced HBV RNA variants is described in Materials and Methods.

^*b*^ There are at least 22 known natural spliced variants of HBV RNAs, Sp1-Sp22 (54–56). Along with those natural splice variants, there were also putative HBV splice variants found in transfected cells, named as pSPs (54). We found pSP10 and pSP12 in our samples. The pSP10 (2/2) indicates that we detected the second deletion related to this spliced variant in RNA reads and recognized it as the pSP10 HBV splice RNA variant, because both splicing-generated deletions of pSP10 were reported to be present together in the same sequence (54).

^*c*^ This indicates that the same splice variant was also found in another sample. There 387 stands for SNU-387, 761 stands for SNU-761, etc., while Ctr stands for Control. Control (shown in the table as Ctr) represents RNA isolated from Huh7 cells transfected with the construct pT-HBV1.3 and replicating HBV genome (also shown in the table as Huh7/HBV). It was used as the control for comparison with the material isolated from parental SNU cell lines.

^*d*^ The spliced HBV RNAs previously found by us in serum samples of individuals chronically infected with HBV were indicated as Y (yes) (12).

^*e*^ The positions of the start and end of each spliced sequence are indicated within the reference HBV genome GQ358158 and within the sequence of the actual HBV variant present in the corresponding SNU cell line.

^*f*^ For each spliced HBV RNA variant detected, the percentage of the spliced reads in the total number of reads (spliced and not spliced) covering the spliced area in the sample is shown.

^*g*^ The identified spliced HBV RNA variants that are not identical to previously reported spliced HBV RNAs were also mapped and are considered novel spliced HBV RNAs in this study. Further characterization of these novel spliced variants is warranted.

Both known and novel spliced RNA variants were detected. The splice variant Sp1 (54–56) was found in SNU-886, SNU-761, and in Huh7 cells replicating HBV. Variants Sp14, Sp21, and pSP12 (54–56) were found in SNU-354 and in transfected Huh7 cells. In addition, Sp6, Sp9, Sp11, Sp13, Sp22, and pSP10 (the second segment out of two) (54–56) were observed in transfected Huh7 cells as well. Thus, some of the same splice variants were detected in different cell lines. In addition to known variants, a splicing-associated deletion spanning pos. 2,110–3,215/1–342 was found in SNU-886 and SNU-387 cell lines, while the variant with deletion 124–324 was found in SNU-761 and in transfected Huh7 cells (Table 4). Here and thereafter, we use the numbering of the reference HBV genome GQ358158, while the actual positions in the particular HBV genome variants are provided in Table 4. A total of 16 novel spliced HBV RNA variants were found in five parental SNU cell lines (Table 4).

The population of the spliced HBV-related RNAs in four parental SNU cell lines (SNU-886, SNU-387, SNU-761, and SNU-354) appeared to be relatively abundant, because they bear spliced RNAs, the number of which relative to the total sum of the numbers of reads (spliced and unspliced) crossing the splice start and splice end for a particular spliced area exceeds 50% (Table 4). Furthermore, considering only the most frequent splice variants corresponding to the highest number of unique spliced reads per area of splicing, for SNU-886, this was the novel splice variant with the deletion 1,595–1,694 that constitutes 59.08% of all RNA reads covering the corresponding spliced area. For SNU-387, it was the novel splice variant with the deletion 8–55 (66.67%), for SNU-761, it was the novel splice variant with the deletion 1,644–1,646 (58.62%), and for SNU-354, it was the novel variant with the deletion 1,801–1,847 (96.30%) (Table 4). In fact, in all SNU cell lines shown in Table 4, the most frequent were novel splice HBV RNA variants. The data suggest that novel recurring spliced HBV RNA variants warrant further investigation on their possible functions in the HBV life cycle in follow-up studies.

Interestingly, we previously reported Sp1, pSP12, Sp14, Sp21, Sp13, Sp9, Sp6, pSP12, and a novel variant with the deletion 1,935–3,215/1–928 in the population of HBV RNA species found in sera harvested from individuals chronically infected with HBV (12). Given that our above-described analysis suggested that SNU-739, SNU-387, and SNU-354 have no ongoing HBV genome replication in them, it should be noted that HBV-related splicing events occur there likely on both types of integrant-transcribed HBV RNAs, 5′-human-HBV-3′ RNAs and 5′-HBV-human-3′ RNAs (or id-RNAs), and not on rd-RNAs. This suggests that such splicing takes place during HBV transcription without a need for ongoing HBV genome replication.

### Replication markers of HBV in SNU cell lines transfected with the plasmid initiating HBV genome replication

All the above-mentioned SNU cell lines were transfected with the plasmid pT-HBV1.3, which initiates HBV genome replication (27), to examine if these cell lines derived from HBV-related HCCs can support efficient HBV genome replication. Huh7 cells and HepG2 cells transfected with pT-HBV1.3 served as the controls, because they were derived from HCCs that were unrelated to HBV infection, and they were known to support considerable levels of the HBV genome replication (41, 46, 57, 58). The transfected cells were analyzed on day 8 post-transfection. As mentioned above, we quantified the intracellular accumulation levels of pgRNA, total HBV RNA, rd-RNAs, core-associated DNA, and cccDNA using the same approaches as were employed for the analysis of parental (untransfected) SNU cell lines (see Table 1). The results of the analysis are presented in Table 5, where the levels of HBV markers are expressed as the percentage relative to the levels observed in transfected Huh7 cells. The levels of HBV replication markers observed in Huh7 cells replicating HBV genome were used as 100% values. It became clear that none of the SNU cell lines were able to efficiently support HBV genome replication compared to Huh7 cells and HepG2 cells. The observed inability of SNU cell lines to support efficient HBV replication (Table 5) was consistent with the above-described theory that during chronic HBV infection, livers get repopulated with hepatocytes that poorly replicate HBV and that HBV-associated HCCs often originate from such hepatocytes that have cleared HBV from them or support only very low levels of HBV replication (6–8, 28–32).

**TABLE 5:** Intracellular HBV replication markers in SNU cell lines transfected with pT-HBV1.3

HBV replication markers^*a*^							
SNU cell line	pgRNA% ± SD	Total HBV RNA% ± SD	rd-RNAs% ± SD	Core-associated DNA% ± SD	cccDNA% ± SD	Pattern of HBV replication markers^*b*^	Group^*c*^
SNU-886	1.95 ± 0.31	2.51 ± 0.14	3.15 ± 0.81	0.56 ± 0.17	7.29 ± 2.87	Low pgRNA, total HBV RNA, rd-RNAs, core-associated DNA, and cccDNA	Group 1
SNU-739	0.17 ± 0.02	4.14 ± 0.63	0.59 ± 0.10	0.21 ± 0.08	1.26 ± 0.69		
SNU-387	1.84 ± 0.40	7.34 ± 0.84	8.18 ± 0.57	0.94 ± 0.19	12.03 ± 1.11		
SNU-423	0.23 ± 0.06	0.95 ± 0.26	0.75 ± 0.08	0.63 ± 0.25	5.00 ± 0.53		
SNU-761	27.94 ± 5.97	112.79 ± 15.16	87.20 ± 13.59	2.86 ± 1.19	198.00 ± 19.71	Moderate pgRNA, high total HBV RNA, rd-RNAs, and cccDNA, but low core-associated DNA	Group 2
SNU-475	17.70 ± 7.39	50.57 ± 7.51	68.38 ± 19.44	4.41 ± 1.64	201.97 ± 31.53		
SNU-368	1.49 ± 0.16	9.45 ± 1.91	6.12 ± 0.76	0.27 ± 0.22	37.36 ± 4.01	Low pgRNA, total HBV RNA, rd-RNAs, core-associated DNA, but high cccDNA	Group 3
SNU-354	1.12 ± 0.42	8.54 ± 0.53	5.51 ± 0.96	0.58 ± 0.17	45.62 ± 11.67		
SNU-182	4.16 ± 0.96	10.68 ± 1.26	12.53 ± 2.95	1.35 ± 0.41	77.25 ± 4.90		
SNU-449	3.44 ± 0.90	13.32 ± 1.17	11.44 ± 1.10	2.15 ± 1.51	91.20 ± 17.80		
SNU-398	2.14 ± 0.46	7.29 ± 1.57	6.60 ± 0.94	0.05 ± 0.01	21.92 ± 3.24		
Huh7/HBV	100.00 ± 19.52	100.00 ± 30.23	100.00 ± 14.61	100.00 ± 5.65	100.00 ± 31.70		
HepG2/HBV	65.05 ± 12.04	58.58 ± 3.99	56.78 ± 11.18	76.07 ± 23.82	88.80 ± 1.23		

^*a*^ HBV replication markers are expressed as mean values of the percentage of the levels observed for transfected Huh7 cells replicating HBV genome (Huh7/HBV) plus/minus standard deviation (SD). The levels of HBV replication markers observed for Huh7 cells transfected with pT-HBV1.3 were used as 100% values. HepG2 cells are an additional control cell line, which originated from human HCC not related to HBV infection, and it supports efficient HBV genome replication after transfection with pT-HBV1.3 (HepG2/HBV) similarly to that of Huh7 cells (41, 46, 57, 58).

^*b*^ Patterns reflect the relative accumulation levels of five different intracellular HBV replication markers, indicating the existence of different natural host-mediated mechanisms of suppression of HBV genome replication affecting the levels of each of the markers to a different extent while reducing the overall HBV replication rate in each case.

^*c*^ The SNU cell lines were grouped based on the patterns of accumulation of intracellular HBV markers in post-transfection settings (after SNU cell lines were transfected with the construct pT-HBV1.3 that initiates HBV genome replication). Group 1 includes SNU cell lines, in which we observed low levels of all five markers, pgRNA, total HBV RNA, rd-RNAs, core-associated DNA, and cccDNA (pattern 1). Group 2 includes SNU cell lines, in which we found moderate pgRNA, high total HBV RNA, rd-RNAs, cccDNA, but low core-associated DNA (pattern 2). Group 3 incorporates SNU cell lines that displayed low pgRNA, total HBV RNA, rd-RNAs, core-associated DNA, but high cccDNA (pattern 3).

It needs to be mentioned that the observed differences in the levels of intracellular HBV replication markers in post-transfection settings between the SNU cell lines and Huh7 and HepG2 cells cannot be explained by the differences in the transfection efficiencies. The transfection efficiency levels are shown in Fig. S1, where the results were expressed relative to the transfection efficiency of Huh7 cells, which was used as a 100% value. We found that the transfection efficiency for SNU-886, SNU-449, and SNU-398 exceeded 67% compared to that of Huh7 cells. The transfection efficiencies for SNU-761, SNU-739, SNU-475, SNU-423, SNU-354, and SNU-182 were in the range of 93%–193%. Only for SNU-387 and SNU-368, the transfection efficiencies were between 32% and 34% (Fig. S1). The transfection efficiency for HepG2 cells was 29% relative to that of Huh7 cells. As mentioned above, in all SNU cell lines, HBV genome replication was suppressed, and both Huh7 and HepG2 efficiently supported HBV replication (Table 5), while at the same time, 9 of 11 SNU cell lines had their transfection efficiency either similar to or higher than that of Huh7 cells (Fig. S1).

Based on the levels of HBV markers, SNU cell lines can be divided into three distinct groups (Table 5). Group 1 includes SNU-886, SNU-739, SNU-387, and SNU-423. In these cell lines, we found (i) low levels of pre-core/pgRNAs (between 0.17% and 1.95% compared to Huh7 cells), (ii) low numbers of total HBV RNA (0.95%–7.34%), (iii) low accumulation of rd-RNAs (0.59%–8.18%), (iv) low levels of core-associated DNA (0.21%–0.94%), and also (v) low numbers of cccDNA (1.26%–12.03%). Group 2 includes SNU-761 and SNU-475 cell lines. They displayed (i) moderate levels of pre-core/pgRNA (17.70%–27.94%), (ii) low levels of core-associated DNA (2.86%–4.41%), but high numbers of (iii) total HBV RNA (50.57%–112.79%), (iv) rd-RNAs (68.38%–87.20%), and (v) cccDNA (198.00%–201.97%). Only these two cell lines were better than the other SNU cell lines in terms of the accumulation levels of HBV replication markers post-transfection. Group 3 includes SNU-368, SNU-354, SNU-182, SNU-449, and SNU-398, for which we observed low levels of (i) pre-core/pgRNA (1.12%–4.16%), (ii) total HBV RNA (7.29%–13.32%), (iii) rd-RNAs (5.51%–12.53%), and (iv) core-associated DNA (0.05%–2.15%), and (v) moderate/high levels of cccDNA (from 21.92% to 91.20%) (Table 5). Interestingly, all SNU cell lines displayed low levels of core-associated DNA, and only group 1 had low levels of cccDNA, while groups 2 and 3 supported high/moderate levels of cccDNA (Table 5).

**Fig 5:**
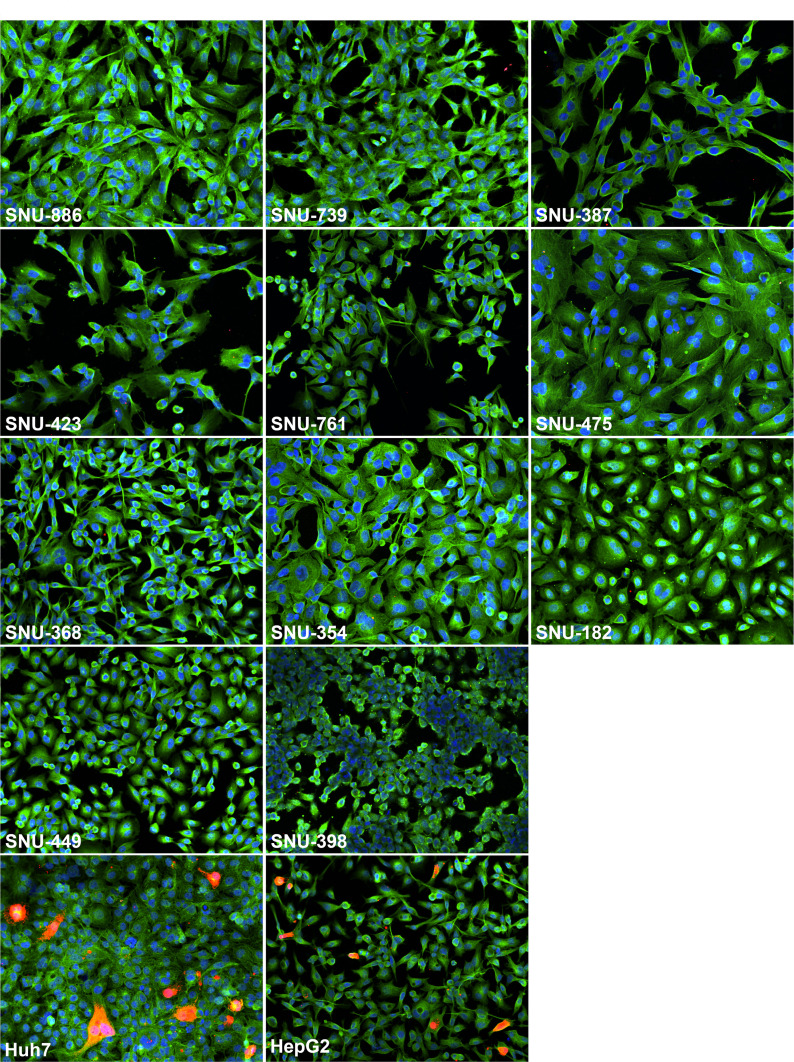
Analysis of HBcAg expression in SNU cell lines transfected with pT-HBV1.3 using immunofluorescence. SNU cell lines, Huh7 cells, and HepG2 cells were transfected with pT-HBV1.3, and then on day 8 post-transfection, they were washed, fixed, permeabilized, and stained for HBcAg (red), beta-tubulin (green), and nuclear DNA (blue) as detailed in Materials and Methods. The names of the cell lines analyzed are indicated on the images.

**Fig 6:**
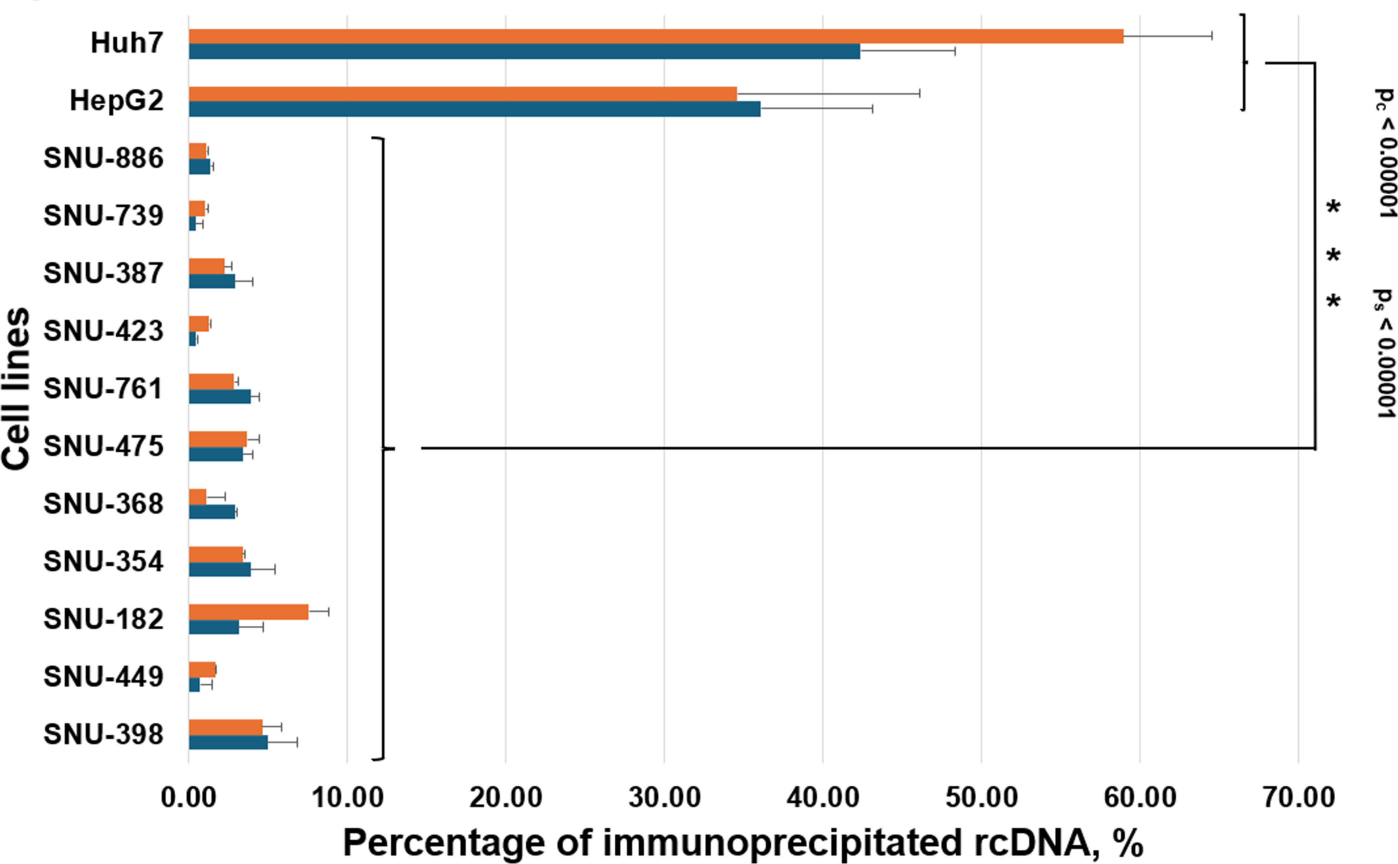
Analysis of secreted HBV DNA associated with unenveloped cores or HBsAg-enveloped virions in SNU cell lines transfected with pT-HBV1.3 using immuno-precipitation. The SNU cell lines, Huh7 cells, and HepG2 cells were transfected with pT-HBV1.3. On day 8 post-transfection, the media were collected from the transfected cells and analyzed via IP either by employing anti-HBcAg antibodies to quantify HBV DNA associated with secreted unenveloped cores or by using anti-HBsAg antibodies to measure the secreted virion-bound HBV DNA as detailed in Materials and Methods. The blue bars represent the data for the virion-associated HBV DNA, while orange bars display the data for the core-bound HBV DNA. The control values for Huh7 and HepG2 cells were the same as for Fig. 3. The data (average value) are presented as the percentage of total level of secreted HBV DNA in the media. We used the Mann-Whitney *U* test and compared two groups of cells: parental SNU cell lines transfected with pT-HBV1.3 versus control Huh7 cells and HepG2 cells that were also transfected with pT-HBV1.3. The test found a statistically significant difference between these two groups of cell lines, and thus, the null hypothesis that there is no difference between these two groups can be rejected. This was anticipated, since only transfected Huh7 cells and HepG2 cells efficiently replicate HBV and secrete considerable levels of core-bound and virion-associated HBV DNA. This was not the case for the transfected SNU cell lines, which were unable to efficiently support HBV genome replication post-transfection and did not secrete any considerable levels of core-bound or virion-bound HBV DNA. The results of the statistical analysis are reflected in the figure (*p*_*c*_ is *P*-value for the IP for HBcAg [analysis of secreted core-bound HBV DNA], *p*_*s*_ is *P*-value for the IP for HBsAg [analysis of secreted virion-associated HBV DNA]).

We also used immunofluorescence to examine the expression of HBcAg in transfected cells. Similarly to parental SNU cell lines (Fig. 2), no HBcAg-positive cells were detected in all SNU cell lines transfected with pT-HBV1.3 (Fig. 5). However, HBcAg-positive cells were found as expected in both Huh7 and HepG2 cells transfected with pT-HBV1.3 and replicating HBV (Fig. 5). Next, the secreted core-bound and virion-bound HBV DNA were assayed in the media of transfected cells. Similarly to parental SNU cell lines (Fig. 3), no significant amounts of core-bound or virion-bound HBV DNA were detected for all 11 transfected SNU cell lines, while considerable levels of core-bound and virion-associated HBV DNA were measured in the media of transfected Huh7 cells and HepG2 cells (Fig. 6). All these findings reflect the inability of SNU cell lines to support efficient HBV replication in post-transfection settings. Overall, considering low levels of intracellular core-associated DNA and undetectable intracellular HBcAg along with a lack of significant levels of secreted core-bound and virion-bound HBV DNA in transfected SNU cell lines, it became apparent that HBcAg likely is a common target for the above-mentioned natural mechanisms suppressing HBV replication. It appears that in SNU cell lines, HBcAg was not produced in sufficient amounts to support HBV genome replication, or the produced HBcAg was efficiently destroyed, and either scenario resulted in suppressed HBV replication.

In summary, the observed three distinct patterns of accumulation levels of HBV markers (Table 5) suggest that there could be at least three natural mechanisms that can efficiently suppress HBV in infected hepatocytes or eliminate the virus from them and that these mechanisms could be facilitated by at least three different expression profiles of host genes, mediating efficient and persistent suppression of HBV replication in the settings of natural chronic HBV infection.

To further elaborate on the point of differential expression profiles, using RNA-seq data, we evaluated the expression levels of a number of human transcription regulators, which were previously suggested to be involved in the regulation of HBV RNAs’ transcription, and of a few other factors that could be involved in mediating the functions controlled by the above-mentioned transcriptional regulators (59). The results summarized in Table 6 presenting the fold changes in the expression levels of these host factors in parental SNU cell lines versus their levels found in parental Huh7 cells. We also took into consideration that we have not observed any significant changes in the expression levels (in SNU cell lines as compared to Huh7 cells) of transcription factors such as activating transcription factor 2 (ATF2), nuclear transcription factor Y (NF-Y), regulatory factor Box 1 (RFX1), signal transducer and activator of transcription 1 (STAT1), and STAT3, which were implicated in the regulation of HBV transcription (59). Since these transcription factors were not differentially expressed compared to Huh7 cells, they are not included in Table 6. The top half of Table 6 shows the transcriptional regulators that could be involved in the stimulation of HBV transcription and their associated host factors, while its bottom displays the transcriptional regulators that could participate in the inhibition of HBV transcription and their associated factors. Clearly, tumor protein p53 (TP53) and TP53-inducible protein 13 (TP53I13) are unlikely to mediate the suppression of HBV genome replication in SNU cell lines, because they were suggested to act as inhibitors of HBV transcription (59), and they were downregulated in most of SNU cell lines (Table 6). There is a certain uniformity among the potential enhancers of HBV transcription. All these factors were downregulated in practically all SNU cell lines and may, to a certain extent, participate in the suppression of HBV genome replication. The strongest downregulation was found for CCAAT enhancer binding protein alpha (CEBPA), hepatocyte nuclear factor 4 alpha (HNF4A), and Kruppel-like factor 15 (KLF15). They all were greatly downregulated in all SNU cell lines of group 1 (as defined in Table 5), in SNU-475 (group 2), and three of five SNU cell lines of group 3. In addition, the hepatocyte nuclear factor 4 gamma (HNF4G) was greatly downregulated only in three of four SNU cell lines of group 1 and SNU-449 of group 3 (Table 6). Downregulation of these four factors (and especially CEBPA, HNF4A, and KLF15) should be examined in the follow-up studies as possible modes of suppression of HBV transcription. Interestingly, tumor protein p53 regulated apoptosis-inducing protein 1 (TP53AIP1), which potentially could be involved in the inhibition of HBV transcription, was considerably upregulated in all SNU cell lines (Table 6). The remaining possible inhibitors of HBV transcription were not uniformly differentially expressed among the 11 SNU cell lines, with one exception—zinc finger E-box binding homeobox 2 (ZEB2), which has been implicated in the inhibition of HBV RNA transcription (59), was significantly upregulated in all SNU cell lines except for SNU-368. All other inhibitory regulators shown in Table 6 were greatly upregulated at least in SNU-739 and SNU-423 of group 1, in SNU-475 of group 2, and two to four SNU cell lines of group 3 (Table 6). These findings also suggest that the upregulation of the above-mentioned inhibitory regulators may participate in profound suppression of HBV genome replication in the corresponding SNU cell lines. Overall, the data in Table 6 suggest that profound suppression of HBV replication associated with SNU cell lines at least in part could be mechanistically mediated via regulating HBV RNA transcription.

**TABLE 6:** Differential expression of a selected set of transcriptional regulators and related host factors that could regulate HBV replication

Fold change versus the level of gene expression found in parental Huh7 cells												
Gene name^*a*^	Effect on HBV transcription	Group 1			Group 2			Group 3				
		SNU-739	SNU-423	SNU-387	SNU-886	SNU-475	SNU-761	SNU-398	SNU-182	SNU-449	SNU-368	SNU-354
ENSG00000153879	Enhances	−8.8	−8.6	−13.6	−4.2	−6.1	−2.9	−2.4	−7.6	−4.1	-4	−5.8
(CEBPG, CCAAT enhancer binding protein gamma)												
ENSG00000172216	Enhances	−29.7	−1.6	−1.4	−2.9	−1.2	-1	−2.9	-2	−1.4	−1.5	−3.4
(CEBPB, CCAAT enhancer binding protein beta)												
ENSG00000221869	Enhances	−13.1	−7.3	−2.3	−7.1	-3	−3.5	−7.2	−2.4	−2.2	−3.2	−5.2
(CEBPD, CCAAT enhancer binding protein delta)												
ENSG00000245848	Enhances	−745.3	−3,483.2	−115,279	−5.2	−149.9	−6.8	−15	−92.1	−125.1	−50.8	−86.4
(CEBPA, CCAAT enhancer binding protein alpha)												
ENSG00000163884	Enhances	−5.9	−97.3	−16.9	−54.8	−45.6	−10.3	−61.7	−37.9	−30.3	−25.3	−5.5
(KLF15, Kruppel like factor 15)												
ENSG00000101076	Enhances	−3,018.4	−81,954.5	−10,066.6	−53.2	−1,611	−2.2	−8,516	−1,089.2	−8,720.7	−4.9	-4
(HNF4A, hepatocyte nuclear factor 4 alpha)												
ENSG00000164749	Enhances	−53.3	−54.7	−4,876.1	−1.6	-2	−3.7	−2.6	−6.3	−24.3	−1.9	−1.9
(HNF4G, hepatocyte nuclear factor 4 gamma)												
ENSG00000120471	Inhibits	50.1	24.2	23.4	8.6	25	8.7	277.3	16.9	49.1	16.3	16.5
(TP53AIP1, Tumor protein p53 regulated apoptosis inducing protein 1)												
ENSG00000141510	Inhibits	1.1	1.2	−6.9	−33.6	−1.5	−6.8	−2.7	−1.8	−1.3	−13.2	−10.7
(TP53, tumor protein p53)												
ENSG00000167543	Inhibits	−4.3	−148.7	-3	−3.4	−2.2	−1.8	−156.2	−4.7	−2.8	−17	−8.7
(TP53I13, tumor protein p53 inducible protein 13)												
ENSG00000177519	Inhibits	778	93	107	175.5	335.2	−1.1	7.4	-9	-9	4.2	43
(RPRM, reprimo, TP53 dependent G2 arrest mediator homolog)												
ENSG00000183632	Inhibits	303.4	84.2	1	1	249.8	1	525.7	159.5	1	18.4	10.9
(TP53TG3 [includes others], TP53 target 3)												
ENSG00000205456	Inhibits	662.3	428.7	1	1	374.5	8.7	1,043	612.4	1	154.2	89.3
(TP53TG3D [includes others], TP53 target 3D)												
ENSG00000261509	Inhibits	213.9	101.9	1	1	217.9	1	86.9	15.6	1	12.8	1
(TP53TG3B [includes others], TP53 target 3B)												
ENSG00000278848	Inhibits	122.6	42.4	1	1	42.4	1	318.7	155.8	1	-1	1

(TP53TG3F, LOC102723655 [includes others], TP53 target 3 family member F)												
ENSG00000169554	Inhibits	145.6	96.8	154	250.1	166	16.9	631.7	91.1	55.6	−6.9	53.4
(ZEB2, zinc finger E-box binding homeobox 2)												

^*a*^ Shown are the fold changes for the levels of transcriptional regulators and some related host factors as determined in parental SNU cell lines versus parental Huh7 cells. These transcriptional regulators (CCAAT-enhancer binding protein family, Kruppel-like factor 15, hepatocyte nuclear factor 4 alpha, Tumor protein TP53, and zinc finger E-box binding homeobox 2) were previously suggested to be involved in the regulation of HBV transcription and replication (59). Several different CEBP family members were incorporated in Table 6, because CCAAT-enhancer-binding protein family was suggested to enhance HBV transcription. The HNF4G was included as a paralog of HNF4A, which was implicated in stimulation of HBV transcription (59). The factors TP53TG3, TP53TG3D, TP53TG3B, TP53TG3F as well as some other TP53-related host factors were also included, because TP53 was implicated in the binding of Enhancer II and X promoter regions and inhibiting HBV transcription (59), and the above-mentioned TP53-related factors may play a significant role in TP53-mediated signaling. The above-mentioned transcriptional regulators were reported either as enhancers or inhibitors of HBV transcription (59), and their related factors included in Table 6 are placed in the same functional (expected) category as the corresponding transcriptional regulator. The fold change values were calculated based on the RNA-seq data. Above the name of a cell line, the group is indicated to which a particular SNU cell line belongs based on the data and conclusions presented in Table 5.

## DISCUSSION

The cell lines derived from HBV-associated HCCs harvested from individuals chronically infected with HBV are very important assets for conducting experimental studies addressing various aspects of the HBV life cycle. The detailed characterization of such cell lines is essential for understanding what categories of research questions could be resolved by using these HCC-derived cell culture systems. This study analyzed 11 SNU cell lines, SNU-886, SNU-739, SNU-387, SNU-423, SNU-761, SNU-475, SNU-368, SNU-354, SNU-182, SNU-449, and SNU-398, which were developed by the Seoul National University using the HCC tissues obtained from Korean patients who were chronically infected with HBV. These cell lines were not examined in detail for the HBV markers and the status of HBV replication in them (22–26).

We measured five intracellular markers of HBV genome replication: (i) pre-core/pgRNA, (ii) rd-RNAs (i.e., RNA species transcribed during HBV genome replication from cccDNA and polyadenylated using HBV conventional PAS or replication-derived RNAs), (iii) total HBV RNA (this assay quantifies pre-core RNA, pgRNA, L mRNA, M/S mRNA, S mRNA, X mRNA, and SubX mRNA that were polyadenylated using the conventional or cryptic polyadenylation HBV signal, and it is expected to detect rd-RNAs, cps-RNAs [RNAs bearing only HBV sequences and polyadenylated using cryptic HBV PAS], and also various RNA species derived from integrated HBV DNA depending on the HBV sequences that they will contain), (iv) core-associated HBV DNA, and (v) cccDNA, in parental SNU cell lines and in SNU cell lines transfected with pT-HBV1.3 for initiating HBV genome replication. We also examined the expression of intracellular HBcAg by immunofluorescence and analyzed the secreted core-bound and virion-associated HBV DNA in parental and transfected SNU cell lines using IP. In addition, we also analyzed in detail HBV-related RNA species accumulated in parental SNU cell lines using RNA-seq based on poly(A) selection.

Based on undetectable core-associated DNA and cccDNA in SNU-423 and SNU-368, and undetectable rd-RNAs in SNU-398 (Table 1), these three cell lines were evaluated as the ones without ongoing HBV replication in them. We have considered that the signal measured by the above-mentioned RT-qPCRs for pre-core/pgRNA, rd-RNAs, and total HBV RNA could come from HBV genome replication or from RNAs transcribed from integrated HBV DNA independently of viral replication. When such RNAs are transcribed from integrated HBV DNA, they will be amplified by the assays designed to amplify RNA species produced by the HBV genome replication, because they bear the HBV sequences that are expected to be amplified. The copy number determined by RT-qPCRs in this case depends on the structure and abundance of the HBV DNA integrants, the strength of relevant HBV and human promoters in the context of human chromosomes, the availability of the target sequences, and the way how various transcriptional regulatory mechanisms influence the production of the integrant-transcribed RNAs. It should not be expected that the ratio between the copy numbers measured for the integrant-transcribed RNAs by certain RT-qPCRs targeting different amplicons and tailored to amplify RNAs produced by HBV genome replication should resemble the ratio measured for the RNA species generated by HBV genome replication. At the same time, the signal measured by qPCRs for core-associated DNA and cccDNA could come from HBV genome replication or could come from the sequences of the chromosomal fragments that bear integrated HBV DNA and were present in the DNA preparations that were analyzed, since it is known that the DNA isolation procedures that are used for analyzing core-associated DNA and cccDNA do not guarantee the absence of fragmented chromosomal DNA in the final DNA preparations (42–45). Again, the outcomes of the analysis of such qPCRs that are designed to amplify HBV DNA replication markers will depend on the availability of the target amplicon sequences on HBV DNA integrants and on the abundance of the amplicon-bearing integrated HBV sequences in the sample. It needs to be taken into consideration that when HBV replication is occurring, then the HBV replication markers (DNA and RNA) are the majority of the species of interest present in the cells bearing replicating HBV genomes. In this situation, the input of the HBV sequences present on integrated HBV DNA in the context of cellular genomic DNA, which is present in the final DNA preparation that is being analyzed, is negligible if any. However, when there is no ongoing HBV replication, then sensitive qPCRs will pick up only the material that is available from integrated HBV DNA. Therefore, the measured levels of DNA and RNA replication markers of HBV in the above-mentioned three SNU cell lines, SNU-423, SNU-368, and SNU-398, do not reflect ongoing HBV genome replication (Table 1). The RNA-seq analysis of the HBV RNA reads coverage of the selected regions present only on pre-core/pgRNA and in the region of the HBV pos. 1,831–1,931, which is expected to be present almost exclusively on rd-RNAs (Table 2), was consistent with the absence of HBV genome replication in SNU-423, SNU-368, and SNU-398. It also strongly suggested that six additional SNU cell lines, SNU-182, SNU-449, SNU-475, SNU-354, SNU-387, and SNU-739, do not have ongoing HBV replication in them as well. Thus, only two remaining cell lines, SNU-886 and SNU-761, could still have some residual HBV replication in them, as we observed HBV RNA reads coverage in all areas corresponding to the unique sequences present only on pre-core/pgRNA, which are produced only by viral replication, and a considerable number of reads corresponding to the rd-RNA-specific region (Table 2). These conclusions again suggest that the numbers measured by RT-qPCRs or qPCRs used for the analysis of the above-mentioned HBV RNA and DNA markers, respectively, in 9 of 11 of the above-mentioned parental SNU cell lines (Table 1) reflect RNAs transcribed from integrated HBV DNA independently of HBV replication and integrated HBV DNA sequences present on the fragments of chromosomal DNA. Furthermore, no HBcAg expression was detected by the immunofluorescence in all parental SNU cell lines (Fig. 2), and no significant levels of secreted core-bound and virion-bound HBV DNA were measured in the media collected from parental SNU cell lines (Fig. 3). These findings again support the absence of HBV genome replication in 9 of 11 above-mentioned SNU cell lines and are also consistent with the well-suppressed or absent HBV replication in SNU-886 and SNU-761 cell lines. Importantly, the outcomes of the analysis of the locations of polyadenylation sites within HBV sequence, which was also performed using RNA-seq data (Table S2), did not contradict the above-described interpretations.

Further analysis of RNA-seq data (Table 2) confirmed the potential of the presence of mRNAs for the HBV envelope proteins and X/SubX mRNAs (Fig. 1) in several parental SNU cell lines. This is consistent with the accumulation of HBV integrant-derived 5′-HBV-human-3' RNAs (id-RNAs) bearing complete ORFs for the envelope proteins or complete or truncated in 3′-end area ORFs for X/SubX (9, 34) for those SNU cell lines that do not have ongoing HBV genome replication in them. However, for SNU-761 and SNU-886, there could possibly be not only such 5′-HBV-human-3′ RNAs accumulated but also mRNAs produced by the viral replication. The data are certainly consistent with the notion that during chronic HBV infection, considerable amounts of HBsAg are translated from id-RNAs independently of HBV replication (9–11). The follow-up studies may attempt a full-length RNA sequencing approach to get a better understanding of the repertoire of HBV-related RNA species of different origins that are produced in HCC-derived SNU cell lines. The full-length RNA sequencing could also be useful as an independent line of evidence for the analysis of the HBV genome replication status in SNU cell lines.

Next, we analyzed the accumulation of both types of HBV integrant-transcribed RNA species, 5′-human-HBV-3′ RNAs and 5′-HBV-human-3′ RNAs (id-RNAs), by employing hybrid HBV-specific RNA reads bearing the virus and human sequences at the same time, which were generated by RNA-seq and were found for SNU-886, SNU-739, SNU-387, SNU-761, SNU-368, SNU-354, SNU-182, and SNU-449 (Table 3). Both 5′-human-HBV-3′ RNAs and id-RNAs were found in six SNU cell lines, SNU-886, SNU-739, SNU-387, SNU-761, SNU-368, and SNU-449 and in control Huh7 cells transfected with pT-HBV1.3 and replicating HBV genome (Table 3; Fig. 4). In SNU-182, only id-RNAs were detected and not 5′-human-HBV-3′ RNAs, while in SNU-354, we found only 5′-human-HBV-3′ RNA species. The positions of HBV integrations in SNU cell lines were predominantly random and occurred in various locations on 13 different human chromosomes. For most cases, the integrated viral DNA inserts producing HBV integrant-transcribed RNAs were not located in the coding sequences, i.e., not in the exons, which was consistent with our previous report that examined paired human liver and HCC tissues from untreated individuals chronically infected with HBV (9). For SNU cells, most RNA-generating HBV integrants were in intronic and intergenic regions. The reason for this observed tendency was not immediately apparent and could be an interesting subject for follow-up studies. Interestingly, a number of 5′-human-HBV-3′ RNA species were likely transcribed from integrated DSL HBV genomes, which were missing HBV sequences from the left-hand side (Table 3; Fig. 1). According to the mapped positions of the right-hand side of HBV integrants and considering the HBV RNA reads coverage data (Tables 2 and 3), several SNU cell lines, SNU-886, SNU-761, SNU-739, and SNU-368, could likely be used as experimental model systems for examining in detail the biogenesis and properties of id-RNAs encoding HBV envelope proteins (assuming that we were dealing with integrated full-length DSL HBV genome and considering that HBsAg ORFs end at pos. 835 of the reference sequence [34]). Furthermore, such id-RNAs could be analyzed in the absence of ongoing HBV genome replication at least in SNU-739 and SNU-368 cell lines, which additionally supports the value of SNU cell lines as important model systems for addressing a number of important aspects of the HBV life cycle. It also needs to be mentioned that in four of seven cases when id-RNAs were found in parental SNU cell lines, the number of id-RNA transcripts exceeded the number of RNA reads potentially corresponding to rd-RNAs, and in the remaining two of three cases, we also found considerable levels of id-RNAs compared to rd-RNA numbers in the same SNU cell lines (Tables 2 and 3), which is again consistent with the natural abundance of id-RNAs during chronic HBV infection, and with the notion that a considerably large portion of HBsAg molecules is translated from id-RNAs independently of HBV genome replication (9–11, 13, 14).

RNA-seq data also allowed us to examine the splicing events that took place in HBV-related RNA species, which occurred in SNU-886, SNU-739, SNU-387, SNU-761, and SNU-354 cell lines (Table 4), for which we detected between two and seven different spliced HBV RNA variants per cell line. The smallest splicing-generated deletion was 30 nts, while the largest deletion was 2,593 nts. Both known and novel splice variants were observed. Thus, the known splice variants Sp1, Sp14, Sp21 (or pSP9), and pSP12 (53–55), as well as an additional 16 novel spliced HBV RNA variants were observed in parental SNU cell lines (Table 4). We also previously reported the presence of Sp1, Sp14, Sp21 (or pSP9), and pSP12 (which were detected in SNU cell lines in this study) in sera collected from individuals chronically infected with HBV (12). Interestingly, novel spliced HBV-related RNAs were considerably abundant in SNU-886, SNU-387, SNU-761, and SNU-354 cell lines (Table 4). Since SNU-387, SNU-354, and SNU-739 were evaluated above as cell lines without ongoing HBV genome replication, splicing occurs within the HBV sequences of the hybrid virus-host RNA species transcribed from integrated HBV DNA independently of HBV genome replication. The splice variants in these cell lines do not represent pgRNA or any other rd-RNAs of HBV. Therefore, these SNU cell lines represent valuable model systems for detailed analysis of the mechanism of RNA splicing that takes place within HBV sequences of integrant-transcribed HBV RNA species, which contain both the viral and human sequences, in the absence of HBV genome replication.

In addition, we demonstrated that all 11 SNU cell lines were defective in terms of the support of HBV genome replication in the post-transfection settings compared to Huh7 cells, which supported efficient HBV replication after transfection with the plasmid pT-HBV1.3 (Table 5). The results support the hypothesis that during chronic HBV infection, livers get repopulated with hepatocytes poorly replicating HBV. This persistent and deep suppression of HBV is likely mediated by unique expression patterns of certain host genes. Such hepatocytes, poorly supporting or not supporting HBV genome replication, often become precursors of malignant cells, giving rise to HCCs. HCCs often have very low/undetectable levels of HBV replication (6–8, 28–32), very likely reflecting the unique gene expression profiles of hepatocytes from which HCCs were originated. The observed suppression of HBV replication is apparently mediated by more than one host-mediated mechanism, as suggested by three observed unique patterns of accumulation of intracellular HBV replication markers in transfected SNU cell lines: (i) low levels of pgRNA, total HBV RNA, rd-RNAs, cccDNA, and core-associated DNA; (ii) moderate pgRNA, high total HBV RNA, rd-RNAs, and cccDNA, but low core-associated DNA; and (iii) low pgRNA, total HBV RNA, rd-RNAs, and core-associated DNA, but moderate/high cccDNA. The first pattern was found in SNU-886, SNU-739, SNU-387, and SNU-423 (group 1 of SNU cell lines). The second pattern was observed for SNU-761 and SNU-475 (group 2), and the third pattern was identified for SNU-368, SNU-354, SNU-182, SNU-449, and SNU-398 (group 3) (Table 5). It is quite possible that there will be similar expression patterns of certain key host factors within each group of SNU cells correlating with the observed pattern of HBV markers. Thus, the host-mediated mechanisms of suppression of HBV genome replication apparently affect the accumulation levels of HBV replication markers in a different manner. However, importantly, all HBV markers patterns displayed low core-associated DNA levels, which could indicate that the host most frequently and successfully restricts HBV replication via suppressing the viral reverse transcription (RT) step and RT-related events (Table 5). Furthermore, the above-described findings were also confirmed by the undetectable intracellular HBcAg in transfected SNU cell lines (Fig. 5) and by the lack of considerable levels of secreted core-bound or virion-associated HBV DNA in media collected from transfected SNU cell lines (Fig. 6). Therefore, our data collectively suggest that HBcAg is likely a common major target for the natural mechanisms suppressing HBV genome replication during chronic HBV infection. It became apparent that in transfected SNU cell lines, HBcAg was not produced in sufficient amounts to facilitate HBV replication or the generated HBcAg was efficiently destroyed, and either of those scenarios resulted in suppressed HBV replication. Both of those scenarios will also be consistent with the above-mentioned suppression of RT and RT-related events in HBV genome replication.

Next, our analysis of the expression profiles of selected transcription regulators and some other related host factors that could be involved in regulatory pathways affected by those regulators in parental SNU cell lines (Table 6) indicates that natural efficient suppression of HBV genome replication, at least in part, could be mediated by modulating the HBV RNA transcription. In particular, one of the most profoundly downregulated factors in the majority of the tested SNU cell lines was hepatocyte nuclear factor 4 alpha (Table 6). The HNF4A is reported to interact with HBV core promoter, Enhancer I, Enhancer II, and PreS1 region (59–63). It is apparently upregulated during HBV infection (64). The HNF4A is considered an important stimulator of the HBV RNA transcription (59, 65). It has been shown that downregulation of HNF4A in cell culture and in mice resulted in the inhibition of HBV transcription and replication (66), which makes this host factor a promising candidate that could participate in the natural suppression of HBV replication and infection *in vivo*. Another interesting host factor in this respect is the ZEB2, which is reported to bind the HBV core promoter region and inhibit HBV RNA transcription (59). We found this factor to be greatly upregulated in almost all parental SNU cell lines (Table 6), which is consistent with natural suppression of HBV genome replication. These observations suggest that the mechanisms of the natural suppression of HBV replication could be mediated by the loss of or considerably decreased expression of the factors critical for supporting efficient HBV genome replication, or they could be facilitated by the overexpression of some factor or factors that inhibit HBV replication.

In conclusion, since the above-mentioned suppression of HBV genome replication was demonstrated in all tested SNU cell lines in post-transfection settings (Table 5), and the absence of ongoing HBV genome replication was found in 9 of 11 analyzed parental SNU cell lines (Tables 1 and 2) in the absence of any components of the adaptive immune response, there could be a possibility that chronic HBV infection could be profoundly and persistently suppressed or perhaps even cleared by means of specific gene expression patterns of one or several factors that could be attributed to the host innate immune response, and to achieve that, the restoration of exhausted adaptive immune response may not be needed. We also think that the identification and verification of the host factors (including the upregulated inhibitors of HBV replication and downregulated supporters of HBV replication) involved in mediating natural suppression of HBV genome replication will be useful for advancing our understanding of the mechanisms of the HBV-host interactions and also could be informative for the search for novel anti-HBV interventions.

## MATERIALS AND METHODS

### Transfection

Huh7 cells, HepG2 cells, and SNU cell lines were transfected with the plasmid construct pT-HBV1.3 to initiate HBV genome replication (27) using Lipofectamine 2000 (Invitrogen) according to the manufacturer’s instructions. The cells were harvested for further analysis on day 8 post-transfection.

### RNA isolation for RT-qPCR analysis

Total RNA was isolated from transfected or untransfected (parental) cells using TRI Reagent (Molecular Research Center) according to the instructions of the manufacturer. The isolated total RNA was treated with Turbo DNase (Fisher) for 1 h at 37°C according to the manufacturer’s instructions. DNase-treated RNA (dtRNA) samples were re-extracted with TRI reagent and then used for synthesizing cDNA.

### RNA isolation for RNA-seq analysis

Total RNA from parental (untransfected) SNU cell lines, untransfected Huh7 cells, and Huh7 cells transfected with pT-HBV1.3 was isolated using TRI reagent. Up to 1 µg of extracted total RNA was used further for the generation of individual libraries.

### RT-qPCR to quantify pre-genomic RNA levels

The intracellular accumulation levels of pgRNA were measured as described previously (12). Briefly, isolated and re-extracted dtRNA (0.4 µg) was reverse transcribed using LunaScript RT Master Mix Kit (New England Biolabs) and 4 µM of primer 1288 (2,433-GATTGAGATCTTCTGCGACG-2,414). The generated cDNA was then used for qPCR. The qPCR was performed using TaqMan Gene Expression Master Mix (Applied Biosystems), primers 1286 (2,326-ACTTCCGGAGACTACTGTTGTTAG-2,349) and 1288 (2,433-GATTGAGATCTTCTGCGACG-2,414), and the TaqMan probe 1287 (2,372-6-carboxyfluorescein [FAM]–AGAAGAACTCCCTCGCCTCGCAG-black hole quencher 1 [3BHQ_1]−2,394). The qPCR amplicon covered HBV pos. 2,326–2,433. Here and thereafter, the numbering of the HBV positions was given for the HBV reference genome GQ358158. For calculating the copy numbers of pgRNA, the RNA standard was prepared as described previously (12). Tenfold dilutions of the gel-purified RNA standard were used for the calibration curve, ranging from 2 × 10^2^ to 2 × 10^7^ genome equivalents (GE) of HBV.

### RT-qPCR to measure the levels of rd-RNAs

The intracellular accumulation of rd-RNAs was analyzed as previously described (12). Briefly, up to 0.4 µg of re-extracted dtRNA was used for reverse transcription with LunaScript RT Master Mix Kit and 4 µM of primer 1330b (1,847-AAGAGATGATTAGGCAGAGGTG-1,826). The levels of rd-RNAs were quantified by qPCR using primers 1146b (1,692-GACCTTGAGGCATACTTCAAAG-1,713) and 1330b (1,847-AAGAGATGATTAGGCAGAGGTG-1,826), the TaqMan probe 1157b (1,776-FAM-GGAGGCTGTAGGCATAAATTGGTCTG-3BHQ_1-1,801), and TaqMan Gene Expression Master Mix. The qPCR amplicon spanned the HBV pos. 1,692–1,847. The RNA standard was prepared as described previously (12). To generate the calibration curve, tenfold dilutions of the gel-purified RNA standard were used in the range from 2 × 10^2^ to 2 × 10^7^ HBV GE.

### RT-qPCR to quantify total HBV RNA

Up to 0.4 µg of re-extracted dtRNA was employed for cDNA synthesis using LunaScript RT Master Mix Kit and 4 µM of primer 1996 (1,743-CCAACTCCTCCCAGTCTTTAAAC-1,721). To quantify intracellular levels of total HBV RNA by qPCR assay, the primers 1994 (1,662-ACTCTTGGACTCTCAGCAATGTC-1,684) and 1996 (1,743-CCAACTCCTCCCAGTCTTTAAAC-1,721), the TaqMan probe 1995 (1,689-FAM-ACCGACCTTGAGGCATACTTCAAAGAC-3BHQ_1-1,715), and TaqMan Gene Expression Master Mix were used. The amplified qPCR region covered the HBV pos. 1,662–1,743. The RNA standard for measuring total HBV RNA was produced in the same way as for the quantification of rd-RNAs (12). A series of tenfold dilutions of the gel-purified RNA standard was utilized for the calibration curve, ranging from 2 × 10^2^ to 2 × 10^7^ GE of HBV.

### Isolation and quantification of intracellular core-associated HBV DNA

The intracellular core-associated HBV DNA was isolated as described previously (67) with minor modifications. The cells of SNU cell lines or Huh7 cells or HepG2 cells (parental or transfected) were lysed in NP-40 lysis buffer (50 mM Tris-HCl [pH 8.0], 1 mM EDTA, 1% NP-40, and 1× ReadyShield protease inhibitor cocktail [Sigma]). After the removal of the nuclei and cell debris by centrifugation, the cytoplasmic lysate (supernatant) was incubated with micrococcal nuclease (MNase) (Roche) (150 units/mL) and CaCl_2_ (5 mM) at 37°C for 90 min to degrade the nucleic acids outside NCs (HBV nucleocapsids or cores). The MNase was then inactivated by the addition of 10 mM EDTA. Thereafter, sodium dodecyl sulfate (SDS) (0.5%) and proteinase K (0.6 mg/mL) were used to digest proteins and disrupt NCs and viral DNA-protein complexes. HBV DNA released from NCs was then extracted and purified by the phenol-chloroform extraction procedure and ethanol precipitation. The primers 1320 (2,255-TTGGT[G/C]TCTTTCGGAGTGTGG-2,275), 1323 (2,407-TTGAGA[C/T]CTTCGTCTGCGAG-2,388), the TaqMan probe 1322 (2,306-FAM-AATGCCCCTATCCTATCAACACTTCCG-3BHQ_1-2,332), and TaqMan Gene Expression Master Mix were used to perform the qPCR assay for the quantification of intracellular core-associated HBV DNA levels. The qPCR amplicon spanned the HBV pos. 2,255– 2,407. Tenfold dilutions of the plasmid pT-HBV1.3 were used for the calibration curve, ranging from 1 to 10^7^ GE of HBV.

### Isolation and quantification of intracellular covalently closed circular DNA of HBV

The cccDNA was extracted as described previously (9) with some modifications. Briefly, the cells of SNU cell lines or Huh7 cells or HepG2 cells (parental or transfected) were lysed in SDS lysis buffer (10 mM Tris-HCl [pH 7.6], 10 mM EDTA, 0.5 M NaCl, and 0.5% SDS). After incubation for 30 min at room temperature, the mixture was centrifuged for 30 min at 4°C, and the resulting supernatant was subjected to phenol-chloroform extraction. The precipitated DNA was washed with ethanol, air-dried, and re-suspended in DNase/RNase-free water. The resulting DNA (1 µg in total) was digested with Exonuclease V (New England Biolabs) in 50 µL reaction mixture containing 1× NEB buffer, 1 mM ATP, and 10 U of Exonuclease V. The reaction mixture was incubated for 30 min at 37°C. The reaction was then stopped by adding 10 mM EDTA, and heat inactivation was performed at 70°C for 30 min. Then, cccDNA levels were quantified via qPCR, which used primers 1797 (1,685-AACGACCGACCTTGAGGC-1,702), 1799 (1,946-GTAACTCCACAG[A/T]AGCTCCAAATTCT-1,921), and the TaqMan probe 1798 (1,859-FAM-ACTGTTCAAGCCTCCAAGCTGTGCCTT-3BHQ_1-1,885). To conduct the qPCR assay, TaqMan Gene Expression Master Mix was used. The qPCR amplicon covered the area of the HBV pos. 1,685–1,946. A 10-fold dilution series of the plasmid pCB102 linearized with HindIII was used to generate a calibration curve, ranging from 1 to 10^7^ of HBV GE. The pCB102 plasmid contains a 1.3× HBV genome sequence of genotype B (GenBank accession number D00330). As a control reaction to assess the efficiency of the treatment of circular DNA using Exonuclease V, the plasmid pCB102 (2 ng) was added to untransfected Huh7 cells lysed in SDS lysis buffer and then isolated by phenol-chloroform extraction. The extracted mixture of total cellular DNA and plasmid DNA was then treated with Exonuclease V as described above, and qPCR was then conducted using the primers 1797 and 1799, the probe 1798, and the pCB102 plasmid DNA standard (Table S3). As another control reaction to assess the efficiency of the treatment of linear DNA with Exonuclease V, HBV double-stranded linear DNA genome was used. To obtain HBV DSL DNA, the region of the HBV pos. 1,820–3,182/1–1,830 of HBV genotype D (U95551, serotype ayw, length of the genome = 3,182 bp) was amplified by conventional PCR using the pT-HBV1.3 plasmid as a template. The resulting length of DSL was 3,193 bp. Primers used for PCR amplification of DSL were 1804 (1,820-AACTTTTTCACCTCTGCCTAATCATCTCTTG-1,850) and 1805 (1,830-GTGAAAAAGTTGCATGGTGCTGG-1,808). The *in vitro*-synthesized DSL (2 ng) was added to untransfected Huh7 cells lysed in SDS lysis buffer and subjected to the above-described procedure of HBV cccDNA extraction and Exonuclease V digestion. The qPCR assay was then performed to calculate the yield of DSL after treatment using the oligos 1320, 1323, and the probe 1322, and the pT-HBV1.3 plasmid as a DNA standard (Table S3). The isolated HBV relaxed circular DNA (1 × 10^6^ GE) was used as another control for the efficiency of treating partially double-stranded HBV DNA with Exonuclease V. The HBV rcDNA was isolated from the media of Huh7 cells transfected with the plasmid pT-HBV1.3, then added to untransfected Huh7 cells lysed in SDS lysis buffer, extracted by phenol-chloroform, and digested using Exonuclease V as described above. For qPCR quantification, the primers 1320 and 1323, the probe 1322, and the pT-HBV1.3 plasmid DNA standard were utilized (Table S3).

### RNA sequencing

RNA-seq was conducted on individual libraries generated using total RNA isolated from parental SNU cell lines or from transfected Huh7 cells on the Illumina NovaSeq 6000 Sequencing System at the Genomics Core of the University of Kansas Medical Center. For library preparation, the TECAN Universal Plus stranded (fr-secondstrand) mRNA-Seq Kit was used, and sequencing was conducted with 100-cycle paired-end reads. Each sample yielded between 33.8 and 42.8 million reads, with a median of 38.5 million reads. Quality assessment with the FastQC software (68) showed that all samples had an average Phred score above 30. Sequencing reads were trimmed with TrimGalore (69) to remove low-quality and spurious sequences. The cleaned reads were then aligned to the combined current standard human reference genome assembly (GRCh38) and HBV reference genome (GQ358158) using the STAR software (70), achieving an average mapping rate to the combined genome of 96%. Data analysis, identification, and quantification of reads containing only HBV sequences, reads covering the 1,831–1,931 area characteristic to rd-RNAs, 5′-HBV-human-3′ transcripts (id-RNAs), and 5′-human-HBV-3′ RNAs were performed as previously described (12).

### HBV genome construction and genotyping

The exact HBV genome sequences and genotypes present in the analyzed samples were initially unknown. We used a systematic approach involving mapping, alignment, and manual curation to construct the distinct HBV genomes and assign the genotypes. Each genome was assembled to match one of the eight HBV genotypes (A–H), allowing for possible single nucleotide variations or short insertions/deletions. Reads mapping to HBV within the combined human-HBV reference genome, along with reads unmapped by the human genome (post-repeat removal), were aligned to all HBV isolates in the Hepatitis B Virus database (71) using the Bowtie2 software (72). For each sample, the HBV isolate with the highest proportion of mapped reads (after deduplication with Picard’s MarkDuplicates tool [73]) was used as the starting reference for the final assembly, and the sample was assigned the corresponding HBV genotype. The reference genome for each sample was refined iteratively through variant calling, modification, and remapping steps. Variants were identified using the FreeBayes software (74), and the reference genome was updated accordingly, with reads remapped to the modified genome until further mapping improvements ceased. Final mapped reads were assembled into contigs with the Trinity software (75), aligned to the refined reference using the MUSCLE multiple sequence aligner (76), and manually curated to resolve inconsistencies. The final consensus genome for each sample retained the structure of the initial reference while incorporating observed nucleotide variations.

### Genome annotation and quantification

The newly constructed HBV genomes exhibited distinct sequence structures reflective of their corresponding parent isolates. To maintain consistency in referencing genomic regions across these genomes, we aligned each one to the common reference HBV genome (GenBank: GQ358158.1), which we routinely used during the analysis (12), and established base positions relative to this standard. All constructed genomes showed high sequence similarity to GQ358158. Insertions relative to this genome were annotated by marking the position of the last matching base before the insertion, followed by the insertion number, separated by a period. Each genome was further annotated based on the regions defined in the GQ358158 reference HBV genome. Reads unaligned to the human genome were mapped to each HBV sample’s reconstructed reference genome using the Bowtie2 software (72). For each annotated region, we quantified mapped reads by counting unique, stranded reads spanning the region in the deduplicated alignment file, using bioinformatics tools within MATLAB. This count was calculated for each base within the region, summed, and then normalized by the region’s length to yield an average read count per base.

### Identifying viral integration events

The reads reflecting the 5′-human-HBV-3′ or 5′-HBV-human-3′ integrant-transcribed HBV RNAs were identified using the Virus-Clip software (77), followed by manual curation. Identified HBV integration events were then used to assemble 5′-HBV-human-3′ and 5′-human-HBV-3′ RNA transcripts, after which reads were remapped to these transcripts to obtain integration site statistics. We used the strand orientation (fr-secondstrand) of reads to establish the 5′–3′ direction of RNA transcripts.

### Identifying spliced HBV RNA variants

HBV RNA splice variants for each sample were identified by mapping reads (that did not align to the human genome) to the reconstructed HBV reference genomes using the STAR software (70) in two-pass mode. Splice junction data were extracted from STAR’s “SJ.out.tab” output file. Splice variants with both canonical and non-canonical splice sites were included. For each splice site, a percent-splice-in (PSI) value was calculated as follows: PSI = (2 × number of uniquely mapped spliced reads)/(number of unique reads crossing splice start + number of unique reads crossing splice end).

### Identifying polyadenylation sites within the HBV sequence

Analysis of the poly(A) sites was mostly done as described previously (12). Briefly, poly(A)-tailed reads were extracted using the Cutadapt software 11 (78), then aligned to the respective HBV reference genomes with Bowtie2 in “sensitive-local” mode. The resulting BAM file was filtered to retain only primary alignments. Strand-specific reads in the alignment were examined for soft clipping at the 3′ end, with the clipped portion checked for poly(A) sequence identity. Soft-clipped sites were reported if poly(A) tails were longer than three bases and had over 80% adenine content.

### Analysis of intracellular HBcAg expression by immunofluorescence

For the analysis of intracellular HBcAg using immunofluorescence, the SNU cells (parental or transfected with the plasmid pT-HBV1.3) were used. As controls, we used HepG2 and Huh7 cells transfected with the plasmid pT-HBV1.3. The transfected cells were analyzed on day 8 post-transfection. Cells were fixed with 4% paraformaldehyde for 15 min at room temperature, washed twice with 1× PBS, and permeabilized with 1× PBS containing 0.2% Triton X-100. Cells were then washed three times with 1× PBS and blocked with 2% bovine serum albumin in 1×x PBS-T (1× PBS containing 0.1% Tween 20). To detect intracellular HBcAg, mouse monoclonal antibody (ab8637, Abcam) was used (1:1,000 dilution). The rabbit monoclonal antibody (4E9G8, Novus Biologicals) was used at a 1:200 dilution to detect beta-tubulin in the analyzed cells. The cells were incubated with primary antibodies for 1 h at 37°C. After being washed three times with 1× PBS-T, the cells were incubated with rhodamine (TRITC)-conjugated goat anti-mouse secondary antibodies and fluorescein (FITC)-conjugated goat anti-rabbit secondary antibodies (Jackson ImmunoResearch) using a dilution of 1:200 for each of the conjugated antibodies for 1 h at room temperature. Then, the samples were washed three times with 1× PBS-T and then stained with 4′,6-diamidino-2-phenylindole (DAPI) (Sigma) at 1 µg/mL for 1 min to detect nuclear DNA. Mounted samples were analyzed using an inverted Eclipse Ti2-E microscope (Nikon), 20× objective, and specific filter blocks, equipped with the ORCA-Fusion BT C15440-20UP camera (Hamamatsu) and running NIS-Elements AR software (Nikon) for acquisition.

### Analysis of the levels of secreted core-bound and virion-associated HBV DNA

To determine the levels of HBV DNA in secreted HBV virions or unenveloped cores in culture media, antibodies against HBsAg (ab9193, Abcam) or against HBcAg (ab8637, Abcam), respectively, were utilized for immunoprecipitation. The media were collected from cultured cells either not transfected (parental) or transfected with the plasmid pT-HBV1.3 on day 8 post-transfection. To precipitate the formed immune complexes, Protein G Dynabeads (Fisher) were used. The fraction of HBV DNA in the immunoprecipitated HBV virions or unenveloped cores was quantified by the above-described qPCR assay for rcDNA (that was used for the analysis of core-associated HBV DNA) via analyzing the pellet and supernatant fractions generated during IP.

## Data Availability

The RNA-seq data have been deposited to NCBI under accession number GSE298154.

## SUPPLEMENTAL MATERIAL

Supplemental material jvi.01144-25-s0001.doc Tables S1 to S3 and Fig. S1.

ASM does not own the copyrights to Supplemental Material that may be linked to, or accessed through, an article. The authors have granted ASM a non-exclusive, world-wide license to publish the Supplemental Material files. Please contact the corresponding author directly for reuse.
