# Supplementary material for: HCC-derived SNU cell lines as model systems to study HBV life cycle
Source: J Virol. 2025 Sep 25;99(10):e01144-25. doi: 10.1128/jvi.01144-25 (PMC12548393; doi:10.1128/jvi.01144-25)
Supplement: Supplemental material — Tables S1 to S3 and Fig. S1. [file jvi.01144-25-s0001.doc]

**Supplementary materials for the manuscript "HCC-derived SNU cell lines as model systems to study HBV life cycle"**

Table S1. Characteristics of HBV consensus genomes identified in parental SNU cell lines.

SNU cell line Number of HBV-specific HBV genotype Size of HBV genome Close match in

RNA reads (nts) NCBI database

(% sequence identity)^c,d^

SNU-886 4558 C 3378 KR013943 (97.61%)

SNU-739 1218 C 3215 GQ475313 (99.19%)

SNU-387 672 C 3215 GQ475308 (98.91%)

SNU-423^a^ 0 ND^b^ ND^b^ ND^b^

SNU-761 38202 C 3303 EU306725 (97.97%)

SNU-475 73 C 3230 AY167091 (99.26%)

SNU-368 1081 C 3215 AP011098 (98.32%)

SNU-354 4280 C 3216 GQ475306 (99.32%)

SNU-182 492 C 3215 AP011098 (96.27%)

SNU-449 453 C 3215 GQ475324 (98.72%)

SNU-398^a^ 0 ND^b^ ND^b^ ND^b^

-------------------------------------------------------------------------------------------------------------------------------------------------------------------

Footnotes

^a^ For SNU-423 and SNU-398 cell lines, no HBV-specific RNA reads were detected by RNA-seq.

^b^ ND, not determined (because no HBV-specific RNA reads were detected by RNA-seq).

^c^ Accession number.

^d^ None of the HBV consensus genomes that were reconstructed using RNA-seq data was the exact match to any of the previously reported full-length HBV genome sequences, which were available in NCBI database.

Table S2. Polyadenylation sites within HBV sequence identified in parental SNU cell lines^a^.

Reference Samples

HBV

genome Control^b^ SNU-886 SNU-739 SNU-387 SNU-761 SNU-354 SNU-449

poly(A) pos. poly(A) Depth poly(A) Depth poly(A) Depth poly(A) Depth poly(A) Depth poly(A) Depth poly(A) Depth

(GQ358158) pos pos pos pos pos pos pos

7 7 1

159 159 1

235 235 1

340 340 1 340 2

576 576 2

630 630 1

770 770 1

805 805 1

844 844 1

858 858 1

863 863 1

870 870 6

871 871 1

927 927 3

929 929 35

949 949 1

964 964 2

1104 1104 1

1126 1126 2

1178 1178 1

1276 1276 1

1277 1277 1

1344 1344 2

1622 1622 1

1623 1623 2

1633 1633 3

1636 1636 1

1679 1679 1

1767 1798 1

1768 1799 1

1789 1789 1

1791 1791 1

1796 1796 1

1800 1800 1

1811 1811 1 1974 1 1898 1 1811 2

1813 1813 1

1814 1814 1

1816 1816 1

1817 1817 1

1818 1818 5

1826 1826 1

1827 1827 1

1849 1849 1

1886 1886 3

1892 1892 2

1917 1917 1

1933 1933 7

1934 1934 188 2022 1

1935 1935 5 2098 1 2023 5 1936 1

1936 1936 237 2099 1 1936 1 2024 67 1937 6

1937 1937 8 2025 2

1938 1938 7 2026 1

1939 1939 2 2102 1 1939 1 2027 1

1940 1940 10

1941 1941 21 2104 1 2029 5 1942 2

1942 1942 2

1943 1943 1

1944 1944 1 1945 1

1945 1945 14 2033 3

1947 1947 4 1948 1

1948 1948 1

1950 2038 1

1951 1951 3

1955 2043 1

2018 2018 1

2048 2048 1

2091 2091 1

2468 2468 3

2563 2563 1

2574 2574 2

2614 2614 1

2616 2616 1

2657 2820 1

2781 2781 3

2924 2922 13

2990 2988 1

2994 2992 1

3153 3151 1

3162 3250 1

3204 3202 1

3213 3301 1

-------------------------------------------------------------------------------------------------------------------------------------------------------------------------------------Footnotes

^a^ Only for SNU-886, SNU-739, SNU-387, SNU-761, SNU-354, and SNU449 cell lines, we recovered sufficient number of HBV-specific RNA reads to conduct the analysis. The approach used for identification of polyadenylation sites is described in Materials and Methods. The depth parameter indicates how many times a particular poly(A) addition site was detected in the specific position within HBV sequence. The positions of poly(A) addition sites in the HBV sequence GQ358158 (reference HBV genome) are shown at the left-hand side, and for each sample, the corresponding pos. in the actual HBV variant is given as well. The area within the pos. ~1800-1827 (in the reference HBV genome) likely represents the poly(A) addition sites facilitated by cryptic HBV PAS. The area spanning the pos. ~1929-1953 (in the reference HBV genome) likely reflects the polyadenylation mediated via conventional HBV PAS (12).

^b^ Control represents RNA isolated from Huh7 cells transfected with the construct pT-HBV1.3 that initiates HBV genome replication. It was used for comparison with the material isolated from SNU cell lines.

**Figure S1.**


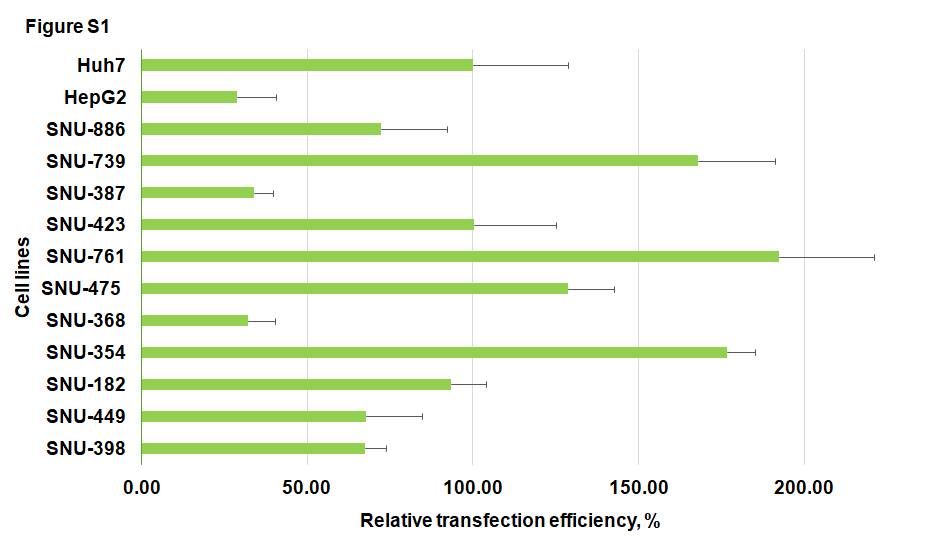


**Figure S1.** **Comparison of the transfection efficiencies between SNU cell lines and Huh7 and HepG2 cells.** Parental SNU cell lines and control Huh7 cells and HepG2 cells were transfected with the plasmid pGFP expressing green fluorescent protein. The transfected cells were analyzed 48 hours post-transfection. The numbers of GFP-positive cells and the total numbers of the cells were acquired by the means of microscopy. The percentage of GFP-positive cells observed for transfected Huh7 cells was used as 100% value of transfection efficiency. The transfection efficiencies for other cell lines were expressed as the percentages relatively to that of Huh7 cells.

Table S3. Examples of the usage of the controls during the analysis of intracellular cccDNA levels^a^

------------------------------------------------------------------------------------------------------------------------------------

Type of the control Percentage of the controls remaining after DNase treatment (%)

------------------------------------------------------------------------------------------------------

Example 1 Example 2 Example 3

------------------------------------------------------------------------------------------------------------------------------------

Plasmid pCB102 83.879+/-2.313 108.692+/-31.565 89.965+/-24.416

(mimics cccDNA)

rcDNA 0.0372+/-0.0011 0.4658+/-0.1127 0.0000+/-0.0000^b^

DSL 0.0031+/-0.0005 0.0794+/-0.0050 ND

------------------------------------------------------------------------------------------------------------------------------------

Footnotes

^a^ Table depicts several examples of the usage of the controls during the analysis of intracellular cccDNA levels. Details are described in the Materials and Methods. The plasmid pCB102 used as the mimic of cccDNA to show the anticipated yield of cccDNA after completion of the isolation procedure, which included the extraction procedure and treatment with Exonuclease V. The rcDNA and DSL were used to show the anticipated remaining levels of the HBV DNA intermediates, which should be predominantly removed after the completion of the isolation/treatment procedure. The data expressed as the percentage relatively to the level of untreated material, which was considered as 100% value (+/- standard deviation). ND, not done.

^b^ In example 3, rcDNA values measured by qPCR were undetermined, which shows complete removal of rcDNA in that case.
